# 5-Phenoxy Primaquine Analogs and the Tetraoxane Hybrid as Antimalarial Agents

**DOI:** 10.3390/molecules26133991

**Published:** 2021-06-30

**Authors:** Somruedee Jansongsaeng, Nitipol Srimongkolpithak, Jutharat Pengon, Sumalee Kamchonwongpaisan, Tanatorn Khotavivattana

**Affiliations:** 1 Centre of Excellence in Natural Products Chemistry, Department of Chemistry, Faculty of Science, Chulalongkorn University, Bangkok 10330, Thailand; somruedee.jan@gmail.com; 2 National Center for Genetic Engineering and Biotechnology (BIOTEC), Pathum Thani 12120, Thailand; nitipol.sri@biotec.or.th (N.S.); jutharat.pen@biotec.or.th (J.P.); sumaleek@biotec.or.th (S.K.)

**Keywords:** primaquine, structure–activity relationship, tetraoxane, hybrid drug, antimalarial activity, heme polymerization inhibition activity

## Abstract

The rapid emergence of drug resistance to the current antimalarial agents has led to the urgent need for the discovery of new and effective compounds. In this work, a series of 5-phenoxy primaquine analogs with 8-aminoquinoline core (**7a**–**7h**) was synthesized and investigated for their antimalarial activity against *Plasmodium falciparum*. Most analogs showed improved blood antimalarial activity compared to the original primaquine. To further explore a drug hybrid strategy, a conjugate compound between tetraoxane and the representative 5-phenoxy-primaquine analog **7a** was synthesized. In our work, the hybrid compound **12** exhibited almost a 30-fold increase in the blood antimalarial activity (IC_50_ = 0.38 ± 0.11 μM) compared to that of primaquine, with relatively low toxicity against mammalian cells (SI = 45.61). Furthermore, we found that these 5-phenoxy primaquine analogs and the hybrid exhibit significant heme polymerization inhibition, an activity similar to that of chloroquine, which could contribute to their improved antimalarial activity. The 5-phenoxy primaquine analogs and the tetraoxane hybrid could serve as promising candidates for the further development of antimalarial agents.

## 1. Introduction

Malaria is a mosquito-borne, life-threatening tropical disease caused by *Plasmodium* parasites. It was estimated by the World Health Organization (WHO) that in 2019, there were over 200 million malaria cases and 409,000 deaths reported globally [1]. To date, there is no effective vaccine available, and the effectiveness of the artemisinin-based combination therapies (ACT) as recommended by the WHO has declined continuously due to the rapid emergence of drug-resistant strains of the parasites. In other word, *P.* *falciparum*, the most life-threatening species, has developed resistance against nearly all drugs [2]. Moreover, even after successful treatment of the blood stage infection, some species such as *P. vivax* could form dormant liver-stage hypnozoites that could be reactivated to cause clinical relapse [3]. Therefore, the development of novel antimalarial agent that is active in both the blood and liver stages is highly in demand.

**Figure 1:**
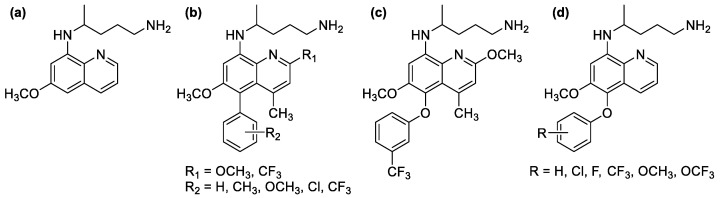
(**a**) Primaquine; (**b**) 5-phenyl-8-aminoquinoline derivatives; (**c**) tafenoquine; (**d**) 5-phenoxy primaquine derivatives.

Primaquine (Figure 1a), the antimalarial drug with 8-aminoquinoline scaffold, is the only drug with radical curative activity against the transient liver forms of *Plasmodium* parasites [4]. However, it has relatively weak schizontocidal activity against *P*. *falciparum* in the blood stage [5]. Typically, compounds in the class of 4-aminaquinoline such as chloroquine are known for their ability to inhibit hematin polymerization as they could bind to the hematin μ-oxo dimer, leading to their antimalarial activity against blood-stage parasites [6]. Nevertheless, despite bearing a similar quinoline core structure, primaquine has a weak heme polymerization activity compared to other compounds in this class [7], which could be related to its lower blood-stage antimalarial activity. Even though the exact mechanism of action of primaquine remains unclear, it was proposed that primaquine could be activated by CPR/CYP2D6 in human liver to produce 5-hydroxylated metabolites, which can stimulate the generation of cellular H_2_O_2_ inducing lethal oxidative stress in malaria parasites via a two-step biochemical relay [8]. However, the formation of the hydroxylated metabolites could cause serious side effects, such as methemoglobinemia and hemolytic anemia in patients with deficiency in glucose-6-phosphate dehydrogenase (G6PD) activity [9], and hence the use of primaquine clinically is discouraged, especially in Southeast Asia where G6PD deficiency is prevalent. It was also later proven that this toxic side effect can be remedied by the installation of substituents at the metabolically labile C-5 position of the 8-aminoquinoline. For example, a series of 5-aryl-8-aminoquinoline derivatives (Figure 1b) was shown to exhibit a greater metabolic stability and significantly less toxicity in rodent studies while retaining the antimalarial activity [10]. Moreover, the structure–activity relationship (SAR) in the study revealed that the substituents on the 5-phenyl ring markedly affects the IC_50_ against *P. falciparum*, where electron-donating groups such as methoxyl or methyl showed better activity than electron-withdrawing substituents. Similarly, tafenoquine (Figure 1c), a recently approved antimalarial drug with a 5-phenoxy-8-aminoquinoline core structure, was also proven to be less toxic and has a longer half-life [11], as well as having greater potency than that of primaquine [12]. However, to the best of our knowledge, the study on the SAR of such a 5-phenoxy-8-aminoquinoline scaffold is relatively lacking. In 1982, a series of 5-phenoxy primaquine derivatives (Figure 1d) was synthesized and tested for their blood schizontocidal antimalarial activity against *P. berghei* infected mice [13]. However, the range of substituents in the study was quite limited, and the direct SAR of these series was difficult to translate due to the distinct pharmacokinetic properties of each compound and other confounding factors in the animal model.

**Figure 2:**
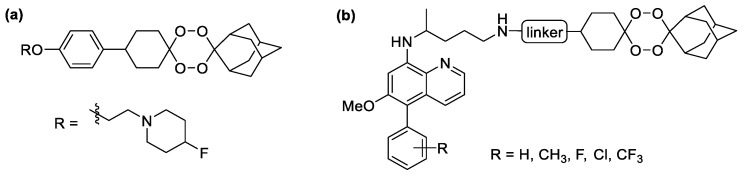
(**a**) E209; (**b**) tetraoxane-5-phenyl primaquine hybrids.

With the rise of drug resistance toward ACTs, hybrid compounds could be a desirable alternative strategy since their ability to hit multiple molecular targets simultaneously could lead to more efficiency toward drug-resistant strains [14]. In particular, novel synthetic peroxide derivative such as E209 (Figure 2a), a clinical candidate tetraoxane analog, was shown to exhibit nanomolar efficacy against multiple strains of *P. falciparum* and *P. vivax*, including the artemisinin-resistant strain [15]. Mechanistically, natural artemisinin, semisynthetic artemisinin, and novel synthetic peroxides require heme activation prior to random alkylation via each radical species [16]. Recently, a range of hybrid compounds between primaquine and tetraoxane was successfully synthesized and were shown to exhibit dual-stage antiplasmodial activity against both the blood stage of *P. falciparum* and liver stage of *P. berghei* with low cytotoxicity toward mammalian cells [17]. Moreover, the metabolic stability of these hybrid compounds was further enhanced by the installation of substituents such as aryls and heteroaryls at the C-5 position of the 8-aminoquinoline (Figure 2b) without the loss of the dual-stage antimalarial activity [18]. In this report, we explore a SAR study of new 5-phenoxy primaquine derivatives toward their in vitro antimalarial activity and cytotoxicity as well as their ability to inhibit hematin polymerization. In addition, the use of 5-phenoxy primaquine as a partner for constructing a tetraoxane hybrid compound as a novel antimalarial agent is also investigated.

## 2. Results and Discussion

### 2.1. Chemistry

**Figure 3:**
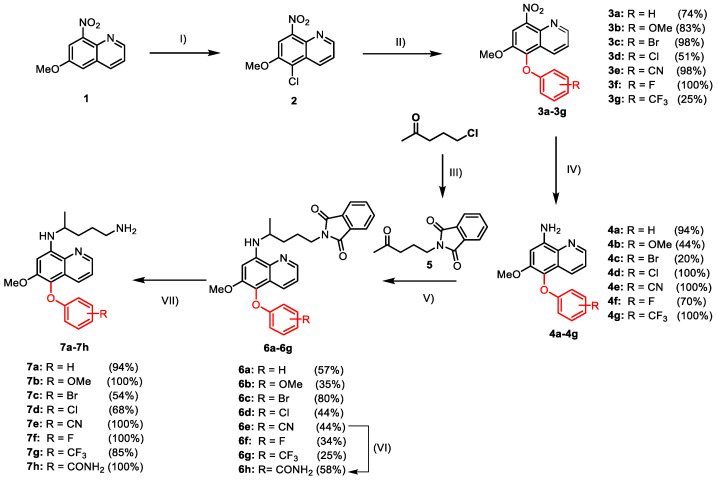
Synthesis of 5-phenoxy primaquine analogs **7a**–**7h**; reagents and conditions: (I) NCS (1.5 equiv.), anh. DMF, 60 °C, 3 h, (80–96%); (II) the corresponding phenols (2.0 equiv.), LiOH·H_2_O (2.0 equiv.), DMSO, 100 °C, 3 h; (III) potassium phthalimide (1.0 equiv.), K_2_CO_3_ (2.0 equiv.), anh. DMF, 80 °C, 3 h (68–70%); (IV) Sn/HCl (10.0 equiv.), EtOH, r.t., 30 min or Pd/H_2_, r.t., 16 h; (V) **5** (5.0 equiv.), NaBH_3_CN (2.0 equiv.), AcOH, anh. MeOH, r.t., 4 days; (VI) 50% H_2_SO_4_, EtOH, 80 °C, 6 h; (VII) NH_2_NH_2_·H_2_O (5.0 equiv.), EtOH, reflux, 30 min.

The synthesis of 5-phenoxy primaquine analogs (Figure 3) started from the chlorination of commercially available 6-methoxy-8-nitroquinoline **1** with *N*-chlorosuccinimide (NCS) [19]. The chlorinated product **2** was used as the key intermediate in the electrophilic aromatic substitution with various phenols, leading to analogs that bear a range of substituents on the 5-phenoxy ring (**3a**–**3g**) in moderate to excellent yields [20]. Next, reduction of the nitro groups on **3a**–**3g** with either Sn/HCl or H_2_/Pd on charcoal gave the amino intermediates **4a**–**4g** which were then undergoing reductive amination with **5** (prepared by the reaction between 5-chloropentan-2-one and potassium phthalimide) under mild conditions to provide **6a**–**6g** in moderate yields [21,22,23]. In addition, the amide derivative **6h** was synthesized by the hydrolysis of the cyanide group from **6e** [24]. Finally, the deprotection of the phthalimide group in **6a**–**6h** led to the desired 5-phenoxy analog of primaquine **7a**–**7h** in excellent yields [10].

**Figure 4:**
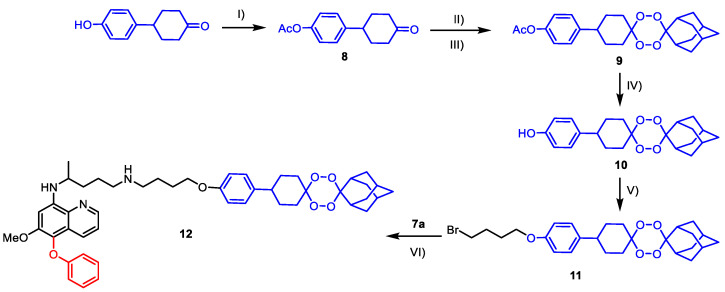
Synthesis of 5-phenoxy primaquine-tetraoxane hybrid (**12**); reagents and conditions: (I) acetic anhydride (3.0 equiv.), Et_3_N (2.0 equiv.), DCM, 0 °C to r.t., (quant.); (II) 30% H_2_O_2_, HCO_2_H/MeCN, 0 °C to r.t.; (III) 2-adamantanone (1.0 equiv.), 5 mol% Bi(OTf)_3_, DCM, r.t., (22% over 2 steps); (IV) LiOH·H_2_O (3.0 equiv.), THF, water, r.t., 3 h, (92%); (V) 1,4-dibromobutane (3.0 equiv.), K_2_CO_3_ (4.0 equiv.), MeCN, 60 °C, 6 h, (73%); (VI) K_2_CO_3_ (1.0 equiv.), KI (0.2 equiv.), anh. DMF, r.t., (51%).

From the result of the antimalarial activity screening as described below in Section 2.2, we picked **7a** as a representative compound for the synthesis of the novel 5-phenoxy primaquine-tetraoxane hybrid (Figure 4). The tetraoxane-OH **10** was synthesized starting from the acylation of a commercially available 4-(4-hydroxyphenyl)cyclohexanone to give **8** [15], which was then converted into the tetraoxane **9** through a two-step process: the generation of the unstable gem di-hydroperoxide intermediate using 30% H_2_O_2_ under acid-catalyzed conditions (Step II), and the reaction with 2-adamantanone in the presence of a catalytic amount of Bi(OTf)_3_ as a Lewis acid (Step III). Basic hydrolysis of the acetate group of **9** provided tetraoxane-OH **10**. In order to connect the tetraoxane with the synthesized 5-phenoxy primaquine, 1,4-dibromobutane was selected as a linker. The alkylation of **10** with was 1,4-dibromobutane gave intermediate **11** in good yield [25]. Finally, the reaction between **11** and **7a** using a catalytic amount of KI gave the desired 5-phenoxy primaquine-tetraoxane hybrid **12** in moderate yield [26].

### 2.2. Blood Antimalarial Activity and Cytotoxicity

The synthesized 5-phenoxy primaquine analogs **7a**–**7h** were screened for their activity against *P*. *falciparum* 3D7 with SYBR^®^ Green assay, and the cytotoxicity against Vero cell with sulforhodamine B (SRB) colorimetric assay (Table 1). Most of the 5-phenoxy analogs, except **7h**, showed a minor increase in the inhibitory activity with the IC_50_ in the range of 3.65 to 8.20 μM compared to that of the original primaquine (IC_50_ = 11.33 μM). Typically, analogs with mesomerically electron-donating group such as methoxy (**7b**) and halogens such as bromo (**7c**) or chloro (**7d**) tend to be less effective than those with strong electron-withdrawing groups such as cyano (**7e**), fluoro (**7f**), and trifluoromethyl (**7g**). Interestingly, the most active compound in the series is the one without any substituents on the 5-phenoxy ring (**7a**), although it should be underlined that the difference in terms of the activity is relatively subtle. This implies that the 5-phenoxy moiety not only enhances the blood-stage activity, but it can also tolerate structural modification, which would be beneficial for fine-tuning the pharmacokinetic/pharmacodynamic properties in further drug development processes. The cytotoxicity against Vero cells showed that all the 5-phenoxy primaquine analogs are less toxic than ellipticine (EPT), an anticancer drug. Analogs **7a**, **7e**, **7f**, and **7h** exhibit comparable cytotoxicity and/or selectivity index (SI) to the PQ while the others process lower SI values. Therefore, the introduction of the 5-phenoxy group led to a slight increase in the cytotoxicity as reference to PQ.

As mentioned earlier, the analog **7a** with the highest activity and acceptable SI was chosen as a representative for the formation of a 5-phenoxy primaquine-tetraoxane drug hybrid **12**. The results were favorable in that **12** exhibited almost a 30-fold greater inhibitory activity than PQ with the IC_50_ values of 0.38 ± 0.11 µM, thus confirming that the introduction of a tetraoxane conjugate partner can significantly enhance the activity. However, the effectiveness of this 5-phenoxy hybrid was slightly lower than the previously reported 5-aryl counterparts, which possessed an IC_50_ as low as 0.015 ± 0.003 µM against blood-stage parasites [18]. Nevertheless, the phenoxy moiety could lead to significant change in the metabolic pathway of the drug, and thus more investigation in this regard is required in the future. In addition, although the hybrid **12** possesses the highest cytotoxicity among all the synthesized compounds, the SI of **12** at 45.61 is much higher than PQ and the others, while the improvement in antimalarial activity of conjugated **12** shows the promising possibility for conjugation between primaquine and synthetic peroxides. Presumably, the inhibitory activities could be mainly from the peroxide moiety as found for other peroxide containing antimalarials [27].

### 2.3. Inhibition of Hematin Polymerization

In blood-stage malarial infection, hemoglobin in erythrocytes is digested by *Plasmodium* parasites into amino acids as their nutrients. During this process, the heme generated as a by-product is typically toxic to the parasites due to its ability to produce free radicals and reactive oxygen species. Hematin polymerization is the major detoxification mechanism that is employed by the parasites, as it converts the toxic free heme into hemozoin via a polymerization process [28,29]. As a result, the ability to inhibit such hematin polymerization is considered as one of the promising mechanisms of action for antimalarial drugs, as commonly seen in compounds in the class of 4-aminaquinoline such as chloroquine. Heme polymerization inhibition activity of all the synthesized compounds was investigated using the protocol reported by Saritha and coworkers (Table 2, the primary data of this study can be found in the Supplementary Materials) [30], and the values obtained for both primaquine (IC_50_ = 319.8 ± 12.0 μM) and chloroquine (IC_50_ = 61.2 ± 1.3 μM) were in good agreement with the literature [30]. However, Vennerstrom and coworkers reported that primaquine was completely inactive with the IC_50_ of over 2500 μM, while the similar range of activity was observed for chloroquine [5]. According to the results, all the 5-phenoxyl primaquine analogs inhibited hematin polymerization more efficiently than did primaquine with the IC_50_ in the range of 101.6–285.8 μM, although the activity did not surpass that of chloroquine. Similar to its blood antimalarial activity, the unsubstituted analog **7a** also exhibited the highest inhibition of hematin polymerization among the series. It was found that the hybrid compound **12** showed comparable inhibitory effect to chloroquine with the IC_50_ of 66.9 ± 3.5 μM, which could be one of the reasons for the significant increase in the blood antimalarial activity of **12** compared to the nonhybrid compounds. This result is in line with a report by Persico and coworkers in 2017, who demonstrated the interaction of plakortin, a natural antimalarial endoperoxide, and its synthetic endoperoxide analog with heme, leading to a production of reactive carbon radical species [31]. The investigation on drug combination and inhibitory activities in other stages of **12** and more to come, especially in liver stage, is further explored.

## 3. Materials and Methods

### 3.1. Chemical Reagents and Instruments

All reagents and solvents were obtained from Sigma-Aldrich (St. Louis, MO, USA), TCI chemicals (Tokyo, Japan), Fluorochem (Hadfield, Derbyshire, UK), and Merck (Darmstadt, Germany). All solvents for column chromatography from RCI Labscan (Samutsakorn, Thailand) were distilled before use. Reactions were monitored by thin-layer chromatography (TLC) using aluminum Merck TLC plates coated with silica gel 60 F_254_. Normal-phase column chromatography was performed using silica gel 60 (0.063–0.200 mm, 70–230 mesh ASTM, Merck, Darmstadt, Germany). Proton, carbon, and proton decoupled fluorine nuclear magnetic resonance (^1^H, ^13^C, and ^19^F{^1^H} NMR) spectra were recorded on a Bruker Advance (III) 400WB spectrometer (Bruker, Billerica, MA, USA) and JEOL JNM-ECZ500/S1 (500 MHz, JEOL, Tokyo, Japan). Chemical shifts were expressed in parts per million (ppm), and *J* values were in Hertz (Hz). High-resolution mass spectra (HRMS) were obtained with a micrOTOF-Q II mass spectrometer (Bruker Daltonics) with electrospray ionization. Lastly, 2-(4-Oxopentyl)isoindoline-1,3-dione (**5**) was synthesized according to the previously reported protocol [22].

### 3.2. Synthesis of 5-Phenoxy Primaquine Analogs ***7a**–**7h***

#### 3.2.1. General Procedure A: Synthesis of Compounds **3a**–**3g**

A solution of **2** in DMSO in a round bottom flask was stirred at room temperature for 15 min. Then, a solution of phenol (1.0 equiv.) and LiOH·H_2_O (1.0 equiv.) in DMSO was added dropwise into a solution of starting material. After complete addition, the reaction mixture was stirred at 100 ºC for 4 h. The reaction was quenched with water, extracted with DCM 3 times and 10% NaOH 3 times. The combined organic layers were washed with brine, dried over anhydrous MgSO_4_, filtered, and concentrated to give the crude product. The crude product was further purified by column chromatography (eluent: EtOAc:hexanes = 1:9 to 1:4) on silica gel to afford the product [20].

#### 3.2.2. General Procedure B: Synthesis of Compounds **4**

A solution of the appropriate 5-hydroxy-8-nitroquinoline analogs **3a**–**3g** (1.0 equiv.) and absolute ethanol in a round bottom flask was slowly added 12M HCl at 0 °C to prevent an exothermic reaction, and then Sn powder (10.0 equiv.) was added into the reaction. The reaction mixture was then stirred at room temperature for 30 min. After the reaction was complete, the reaction mixture was quenched with 12M NaOH until the solution became neutral (pH = 7). The resulting mixture was filtered through a glass Büchner filter funnel, and the filtrate was then extracted with EtOAc. The combined organic layers were washed with water, dried over anhydrous MgSO_4_, filtered, and concentrated to give the crude product. The crude product was used without further purification [21].

#### 3.2.3. General Procedure C: Synthesis of Compounds **6a**–**6g**

A solution of the appropriate 5-hydroxy-8-aminoquinoline analog **4a**–**4g** (1.0 equiv.) and **5** (5.0 equiv.) were dissolved in anhydrous MeOH in a dry round bottom flask. Then, acetic acid was added into the reaction mixture. After the mixture was stirred for 2 h, NaBH_3_CN (2.0 equiv.) was added to it. The solution was then stirred at room temperature overnight. The mixture was diluted with EtOAc, and washed with water and brine. The combined organic layers were dried over MgSO_4_, filtered, and concentrated under reduced pressure. The crude product was further purified by column chromatography (eluent: EtOAc:hexanes = 1:9 to 1:4) on silica gel to afford the product [32].

#### 3.2.4. General Procedure D: Synthesis of Compounds **7**

Compounds **6a**–**6h** (1.0 equiv.) were dissolved in EtOH in a round bottom flask. Hydrazine monohydrate (5.0 equiv.) was added into the solution and the mixture was heated at refluxed for 1 h. A solid precipitate was observed. Then, the solution was cooled to room temperature and filtered by cotton. The filtrate was concentrated to give the crude product as a viscous oil. The crude product was purified by column chromatography (eluent: 5% to 50% MeOH:CH_2_Cl_2_) on silica gel [10].

*N^4^-(6-Methoxy-5-phenoxyquinolin-8-yl)pentane-1,4-diamine* (**7a**): **7a** was synthesized following General Procedure A using **2** (715.9 mg, 3 mmol), phenol (282.3 mg, 3 mmol), and LiOH·H_2_O (126 mg, 3 mmol) in DMSO (5 mL) to give **3a** as a yellow solid (661 mg, 2.23 mmol, 74% yield). Next, **3a** (450.0 mg, 1.52 mmol) was subjected to General Procedure B using Sn powder (1.8 g, 15.2 mmol) and 12M HCl (10 mL) in EtOH (10 mL) to give **4a** as a brown viscous oil (379 mg, 1.423 mmol, 94% yield). A mixture of **4a** (74 mg, 0.28 mmol) and **5** (323 mg, 1.12 mmol) were subjected to General Procedure C using CH_3_COOH (8 µL, 0.14 mmol), NaBH_3_CN (13.0 mg, 0.2 mmol), and anhydrous MeOH (2.5 mL) to give **6a** as a yellow oil (75 mg, 0.16 mmol, 57% yield). Finally, **6a** (51 mg, 0.1 mmol) was subjected to General Procedure D using hydrazine monohydrate (28 µL, 0.57 mmol) and EtOH (500 µL) to give **7a** as yellow oil (33 mg, 0.09 mmol, 94% yield). ^1^H NMR (500 MHz, CDCl_3_) δ 8.56 (dd, *J* = 4.1, 1.0 Hz, 1H, ArH), 8.07 (dd, *J* = 8.4, 1.2 Hz, 1H, ArH), 7.31–7.24 (m, 3H, ArH), 6.99 (td, *J* = 7.4, 0.7 Hz, 1H, ArH), 6.90 (d, *J* = 8.6 Hz, 2H, ArH), 6.49 (s, 1H, ArH), 6.10 (s, 1H, NH), 3.93 (s, 3H, OCH_3_), 3.72 (s, 1H, CH), 2.85 (t, *J* = 6.7 Hz, 2H, CH_2_), 1.84–1.65 (m, 4H, CH_2_), 1.37 (d, *J* = 6.3 Hz, 3H, CH_3_); ^13^C NMR (126 MHz, CDCl_3_) δ159.5, 150.5, 144.9, 143.1, 133.8, 129.9, 129.6, 124.7, 124.5, 122.1, 121.5, 115.0, 93.7, 57.1, 48.2, 41.6, 34.2, 28.9, 20.7; HRMS (ESI^+^): *m/z* calcd. for C_21_H_26_N_3_O_2_^+^ [M + H]^+^ 352.2020, found 352.2052.

*N^4^-(6-Methoxy-5-(4-methoxyphenoxy)quinolin-8-yl)pentane-1,4-diamine (**7b**)*: **7b** was synthesized following General Procedure A using **2** (715.9 mg, 3 mmol), 4-methoxyphenol (372.4 mg, 3 mmol) and LiOH·H_2_O (125.8 mg, 3 mmol) in DMSO (5 mL) to give **3b** as a brown solid (809 mg, 2.48 mmol, 83% yield). Next, **3b** (620.0 mg, 1.90 mmol) was subjected to General Procedure B using Sn powder (2.2 g, 19.0 mmol) and 12M HCl (15 mL) in EtOH (15 mL) to give **4b** as a green solid (248 mg, 0.84 mmol, 44% yield). A mixture of **4b** (88 mg, 0.30 mmol) and **5** (346.8 mg, 1.50 mmol) were subjected to General Procedure C using CH_3_COOH (8 µL, 0.15 mmol), NaBH_3_CN (16.0 mg, 0.3 mmol), and anhydrous MeOH (2.5 mL) to give **6b** as a yellow oil (53 mg, 0.10 mmol, 35% yield). Finally, **6b** (53 mg, 0.1 mmol) was subjected to General Procedure D using hydrazine monohydrate (26 µL, 0.52 mmol) and EtOH (500 µL) to give **7b** as yellow oil (41 mg, 0.10 mmol, 100% yield). ^1^H NMR (500 MHz, CDCl_3_) δ 8.53 (dd, *J* = 4.1, 1.6 Hz, 1H, ArH), 8.05 (dd, *J* = 8.4, 1.5 Hz, 1H, ArH), 7.25 (dd, *J* = 8.5, 4.1 Hz, 1H, ArH), 6.78 (q, *J* = 9.3 Hz, 4H, ArH), 6.44 (s, 1H, ArH), 6.05 (s, 1H, NH), 3.90 (s, 3H, OCH_3_), 3.73 (s, 3H, OCH_3_), 3.68 (dd, *J* = 11.9, 5.9 Hz, 1H, CH), 2.79 (t, *J* = 6.8 Hz, 2H, NH_2_), 1.82–1.61 (m, 4H, NH_2_), 1.34 (d, *J* = 6.3 Hz, 3H, CH_3_); ^13^C NMR (126 MHz, CDCl_3_) δ154.3, 153.6, 150.6, 144.9, 143.0, 133.9, 129.9, 125.1, 124.8, 122.0, 115.6 (2C), 114.7 (2C), 93.8, 57.2, 55.8, 48.2, 41.7, 34.2, 29.1, 20.7; HRMS (ESI^+^): *m/z* calcd. for C_22_H_28_N_3_O_3_^+^ [M + H]^+^ 382.2125, found 382.2161.

*N^4^-(5-(4-Bromophenoxy)-6-methoxyquinolin-8-yl) pentane-1,4-diamine(**7c**)*: **7c** was synthesized following General Procedure A using **2** (715.9 mg, 3 mmol), 4-bromophenol (519.0 mg, 3 mmol) and LiOH·H_2_O (126.0 mg, 3 mmol) in DMSO (5 mL) to give 6-methoxy-5-(4-bromophenoxy)-8-nitroquinoline (**3c**) as a brown solid (1.1 g, 2.93 mmol, 98% yield). A solution of **3c** (638.0 mg, 1.7 mmol, 1.0 equiv.) was dissolved EtOH (20 mL), and 10% Pd on carbon (63 mg, 0.6 mmol, 1.0 equiv.) was added. The mixture was stirred at room temperature under H_2_. After the reaction completed, the Pd was filtered out. The filtrate was then extracted with EtOAc. The combined organic layers were washed with water, dried over anhydrous MgSO_4_, filtered, and concentrated to give **4c** as a yellow solid (117 mg, 0.34 mmol, 20% yield). A mixture of **4c** (69 mg, 0.20 mmol) and **5** (231 mg, 1.00 mmol) were subjected to General Procedure C using CH_3_COOH (5 µL, 0.10 mmol), NaBH_3_CN (10.0 mg, 0.2 mmol), and anhydrous MeOH (1.5 mL) to give **6c** as a yellow oil (88 mg, 0.16 mmol, 80% yield). Finally, **6c** (67 mg, 0.12 mmol) was subjected to General Procedure D using hydrazine monohydrate (30 µL, 0.62 mmol) and EtOH (600 µL) to give **7c** as yellow oil (28 mg, 0.07 mmol, 54% yield). ^1^H NMR (500 MHz, CDCl_3_) δ 8.54 (s, 1H, ArH), 7.98 (d, *J* = 8.0 Hz, 1H, ArH), 7.37–7.22 (m, 3H, ArH), 6.75 (d, *J* = 8.7 Hz, 2H, ArH), 6.43 (s, 1H, ArH), 6.11 (s, 1H, ArH), 3.89 (s, 3H, OCH_3_), 3.68 (s, 1H, CH), 2.78 (s, 2H, CH_2_), 1.86–1.52 (m, 4H, CH), 1.35 (d, *J* = 5.9 Hz, 3H, CH_3_); **^1^**^3^C NMR (126 MHz, CDCl_3_) δ 158.7, 150.4, 144.9, 143.4, 133.7, 132.4 (2C), 129.5, 124.4, 124.0, 122.2, 116.8 (2C), 113.7, 93.2, 56.9, 48.2, 42.1, 34.2, 30.0, 20.7; HRMS (ESI^+^): *m/z* calcd. for C_21_H_25_BrN_3_O_2_^+^ [M + H]^+^ 430.1125, found 430.1148.

*N^4^-(6-Methoxy-5-(4-chlorophenoxy)quinolin-8-yl)pentane-1,4-diamine (**7d**)*: **7d** was synthesized following General Procedure A using **2** (715.9 mg, 3 mmol), 4-chlorophenol (386.0 mg, 3 mmol) and LiOH·H_2_O (125.8 mg, 3 mmol) in DMSO (5 mL) to give **3d** as a yellow solid (509 mg, 1.54 mmol, 51% yield). Next, **3d** (463.0 mg, 1.40 mmol) was subjected to General Procedure B using Sn powder (1.6 g, 14.0 mmol) and 12M HCl (11 mL) in EtOH (11 mL) to give **4d** as a green viscous oil (420 mg, 1.40 mmol, 100% yield). A mixture of **4d** (75 mg, 0.25 mmol) and **5** (289.0 mg, 1.25 mmol) were subjected to General Procedure C using CH_3_COOH (7 µL, 0.125 mmol), NaBH_3_CN (16.0 mg, 0.25 mmol), and anhydrous MeOH (1.5 mL) to give **6d** as a yellow viscous oil (57 mg, 0.11 mmol, 44% yield). Finally, **6d** (56 mg, 0.11 mmol) was subjected to General Procedure D using hydrazine monohydrate (29 µL, 0.57 mmol) and EtOH (700 µL) to give the **7d** as yellow oil (29 mg, 0.075 mmol, 68% yield). **^1^**H NMR (500 MHz, CDCl_3_) δ 8.54 (dd, *J* = 4.2, 1.6 Hz, 1H, ArH), 7.99 (dd, *J* = 8.5, 1.6 Hz, 1H, ArH), 7.30–7.25 (m, 1H, ArH), 7.17 (d, *J* = 9.0 Hz, 2H, ArH), 6.80 (d, *J* = 9.0 Hz, 2H, ArH), 6.43 (s, 1H, ArH), 3.89 (s, 3H, OCH_3_), 3.69 (dd, *J* = 11.8, 5.8 Hz, 1H, CH), 2.79 (t, *J* = 6.1 Hz, 2H, CH_2_), 1.80–1.56 (m, 4H, CH_2_), 1.35 (d, *J* = 6.3 Hz, 3H, CH_3_); **^13^C** NMR (126 MHz, CDCl_3_) δ 158.1, 150.4, 144.9, 143.3, 133.7, 129.6, 129.4 (2C), 126.4, 124.5, 124.2, 122.2, 116.3 (2C), 93.3, 57.0, 48.1, 41.4, 34.1, 20.7; HRMS (ESI^+^): *m/z* calcd. for C_21_H_25_ClN_3_O_2_^+^ [M + H]^+^ 386.1630, found 386.1666.

*4-((8-((5-Aminopentan-2-yl)amino)-6-methoxyquinolin-5-yl)oxy)benzonitrile (**7e**)*: **7e** was synthesized following General Procedure A using **2** (1.4 g, 6 mmol), 4-hydroxybenzonitrile (714.0 mg, 6 mmol) and LiOH·H_2_O (252.0 mg, 6 mmol) in DMSO (10 mL) to give **3e** as a yellow solid (1.9 g, 5.91 mmol, 98% yield). Next, **3e** (358.0 mg, 1.1 mmol) was subjected to General Procedure B using Sn powder (1.3 g, 11.0 mmol) and 12M HCl (8 mL) in EtOH (8 mL) to give **4e** as a black viscous oil (367 mg, 1.26 mmol, 100% yield). A mixture of **4e** (70 mg, 0.24 mmol) and **5** (56.0 mg, 0.24 mmol) were subjected to General Procedure C using CH_3_COOH (40 µL, 0.72 mmol), NaBH_3_CN (15.0 mg, 0.24 mmol), and anhydrous MeOH (2.0 mL) to give **6e** as a yellow viscous oil (54 mg, 0.10 mmol, 44% yield). Finally, **6e** (30 mg, 0.06 mmol) was subjected to General Procedure D using hydrazine monohydrate (30 µL, 0.6 mmol), and EtOH (500 µL) to give **7e** as yellow oil (26 mg, 0.06 mmol, 100% yield). ^1^H NMR (500 MHz, CDCl_3_) δ 8.56 (dd, *J* = 4.2, 1.4 Hz, 1H, ArH), 7.93 (dd, *J* = 8.5, 1.5 Hz, 1H, ArH), 7.53 (d, *J* = 8.7 Hz, 2H, ArH), 7.30 (dd, *J* = 8.5, 4.1 Hz, 1H, ArH), 6.93 (d, *J* = 8.7 Hz, 2H, ArH), 6.42 (s, 1H, ArH), 6.17 (brs, 1H, NH), 3.88 (s, 3H, OCH_3_), 3.69 (s, 1H, CH), 2.79 (t, *J* = 6.8 Hz, 2H, CH_2_NH), 1.82–1.57 (m, 4H, CH_2_CH_2_), 1.35 (d, *J* = 6.3 Hz, 3H, CH_3_); ^13^C NMR (126 MHz, CDCl_3_) δ 162.9, 150.3, 145.0, 143.8, 134.2 (2C), 133.5, 129.1, 124.1, 123.0, 122.5, 119.2, 115.9 (2C), 105.0, 92.5, 56.8, 48.2, 42.1, 34.2, 29.9, 20.7; HRMS (ESI^+^): *m/z* calcd. for C_22_H_25_N_4_O_2_^+^ [M + H]^+^ 377.1972, found 377.1978.

*N^4^-(5-(4-Fluorophenoxy)-6-methoxyquinolin-8-yl) pentane-1,4-diamine (**7f**)*: **7f** was synthesized following General Procedure A using **2** (716.0 mg, 3 mmol), 4-fluorophenol (336.0 mg, 3 mmol) and LiOH·H_2_O (126.0 mg, 3 mmol) in DMSO (5 mL) to give **3f** as a yellow solid (1.0 g, 3.18 mmol, 100% yield). Next, **3f** (628.5 mg, 2.0 mmol) was subjected to General Procedure B using Sn powder (2.4 g, 20.0 mmol), and 12M HCl (14 mL) in EtOH (14 mL) to give **4f** as a black viscous oil (400 mg, 1.41 mmol, 70% yield). A mixture of **4f** (79.5 mg, 0.28 mmol) and **5** (64.7 mg, 0.28 mmol) were subjected to General Procedure C using CH_3_COOH (32 µL, 0.56 mmol), NaBH_3_CN (52.5 mg, 0.84 mmol), and anhydrous MeOH (2.0 mL) to give **6f** as a yellow viscous oil (47 mg, 0.094 mmol, 34% yield). Finally, **6f** (35 mg, 0.07 mmol) was subjected to General Procedure D using hydrazine monohydrate (20 µL, 0.42 mmol) and EtOH (1 mL) to give **7f** as yellow oil (27 mg, 0.07 mmol, 100% yield). ^1^H NMR (500 MHz, CDCl_3_) δ 8.51 (d, *J* = 4.0 Hz, 1H, ArH), 8.01 (d, *J* = 8.4 Hz, 1H, ArH), 7.25 (dd, *J* = 7.7, 3.2 Hz, 1H, ArH), 6.90 (t, *J* = 8.6 Hz, 2H, ArH), 6.79 (dd, *J* = 9.0, 4.2 Hz, 2H, ArH), 6.43 (s, 1H, ArH), 6.03 (brs, 1H, NH), 3.88 (s, 3H, OCH_3_), 3.67 (s, 1H, CH), 2.89 (s, 2H, CH_2_NH), 1.89–1.62 (m, 4H, CH_2_CH_2_), 1.30 (d, *J* = 6.0 Hz, 3H, CH_3_); ^13^C NMR (126 MHz, CDCl_3_) δ 158.7, 156.1 (d, ^1^*J*_CF_ = 177.0 Hz), 150.6, 144.7, 142.7, 125.0, 124.7, 122.2, 116.3 (d, ^3^*J*_CF_ = 7.9 Hz), 115.9 (d, ^2^*J*_CF_ = 15.8 Hz) (4C), 115.8, 94.3, 57.1, 48.1, 40.0, 33.8, 24.6, 20.5; **^19^F{^1^H}** NMR (471 MHz, CDCl_3_) δ −123.31; HRMS (ESI^+^): *m/z* calcd. for C_21_H_25_FN_3_O_2_^+^ [M + H]^+^ 370.1925, found 370.1925.

*N^4^-(5-(4-Fluorophenoxy)-6-methoxyquinolin-8-yl) pentane-1,4-diamine (**7g**)*: **7g** was synthesized following General Procedure A using **2** (716.0 mg, 3 mmol), 4-hydroxybenzotrifluoride (486.0 mg, 3 mmol), and LiOH·H_2_O (126.0 mg, 3 mmol) in DMSO (5 mL) to give **3g** as a pale yellow solid (149 mg, 0.41 mmol, 14% yield). Next, **3g** (149 mg, 0.41 mmol) was subjected to General Procedure B using Sn powder (485 mg, 4.09 mmol) and 12M HCl (3.5 mL) in EtOH (3.5 mL) to give **4g** as an orange viscous oil (95 mg, 0.33 mmol, 82% yield). A mixture of **4g** (91 mg, 0.32 mmol) and **5** (148 mg, 0.64 mmol) were subjected to General Procedure C using CH_3_COOH (38 µL, 0.64 mmol), NaBH_3_CN (20 mg, 0.32 mmol), and anhydrous MeOH (3.0 mL) to give **6g** as an orange viscous oil (22 mg, 0.04 mmol, 13% yield). Finally, **6g** (22 mg, 0.04 mmol) was subjected to General Procedure D using hydrazine monohydrate (20 µL, 0.40 mmol) and EtOH (1 mL) to give **7g** as yellow viscous oil (20 mg, 0.04 mmol, 100% yield). ^1^H NMR (500 MHz, acetone-d_6_) δ 8.72–8.48 (m, 1H, ArH), 8.00 (ddd, *J* = 8.5, 3.5, 1.7 Hz, 1H, ArH), 7.61 (d, *J* = 8.0 Hz, 2H, ArH), 7.41 (ddd, *J* = 8.3, 4.1, 1.5 Hz, 1H, ArH), 7.01 (d, *J* = 8.3 Hz, 2H, ArH), 6.71 (dd, *J* = 16.1, 14.9 Hz, 1H, ArH), 3.91 (dd, *J* = 4.4, 1.7 Hz, 3H, OCH_3_), 3.40 3.17 (m, 2H, CH_2_NH), 2.87 (t, *J* = 7.0 Hz, 2H, CH), 1.94–1.68 (m, 4H, CH_2_CH_2_), 1.35 (d, *J* = 6.3 Hz, 3H, CH_3_); **^13^**C NMR (126 MHz, acetone-d_6_) δ 150.7, 144.8, 144.6, 144.0, 133.4, 131.0, 128.7 (q, ^3^*J*_CF_ = 4.1 Hz), 126.9 (q, ^2^*J*_CF_ = 65.1 Hz), 124.0, 122.9, 122.7 (q, ^1^*J*_CF_ = 265.0 Hz), 122.5, 115.3 (3C), 92.7, 56.1, 50.5, 47.9, 41.2, 34.6, 20.1; ^19^F NMR (471 MHz, acetone-d_6_) δ −61.81; HRMS (ESI^+^): *m/z* calcd. for C_22_H_25_F_3_N_3_O_2_^+^ [M + H]^+^ 420.1893, 420.1939.

*4-((8-((5-Aminopentan-2-yl)amino)-6-methoxyquinolin-5-yl)oxy)benzamide (**7h**)*: A solution of **6e** (890 mg, 1.75 mmol, 1.0 equiv.) was dissolved in 50% H_2_SO_4_ (5 mL) and heated at 80 °C for 6 h. After the reaction is complete, the mixture was neutralized with sat. NaHCO_3_ and extracted with EtOAc. The combined organic layers were washed with water, dried over anhydrous MgSO_4_, filtered, and concentrated. The crude product was further purified by column chromatography (eluent: 1–2% MeOH in DCM) on silica gel to afford **6h** as a yellow viscous oil (541 mg, 1.03 mmol, 58% yield) [24]. Finally, **6h** (461 mg, 0.88 mmol) was subjected to General Procedure D using hydrazine monohydrate (435 µL, 8.8 mmol), and EtOH (5 mL) to give **7h** as yellow oil (204 mg, 0.52 mmol, 59% yield). ^1^H NMR (500 MHz, Methanol-d4) δ 8.51 (dd, *J* = 4.1, 1.4 Hz, 1H, ArH), 8.16 (dd, *J* = 5.7, 3.4 Hz, 1H, ArH), 7.96 (dd, *J* = 11.1, 4.1 Hz, 1H, ArH), 7.77 (s, 1H, ArH), 7.76 (s, 2H, ArH), 7.32 (dd, *J* = 8.4, 4.1 Hz, 1H, ArH), 6.81 (dd, *J* = 8.3, 1.4 Hz, 2H, ArH), 6.58 (d, *J* = 4.1 Hz, 1H, ArH), 3.84 (s, 3H, OCH_3_), 3.82–3.77 (m, 1H, CH), 2.95 (t, *J* = 7.2 Hz, 2H, CH_2_NH), 1.76 (t, *J* = 10.6 Hz, 4H, CH_2_CH_2_), 1.31 (t, *J* = 5.5 Hz, 3H, CH_3_); ^13^C NMR (126 MHz, Methanol-d4) δ 170.5, 162.5, 150.4, 144.8, 143.5, 133.4, 131.7 (2C), 129.3, 129.0, 126.7, 125.6, 124.2, 123.8, 122.1 (2C), 114.4, 93.3, 55.8, 39.6, 33.3, 19.5; HRMS (ESI+): m/z calcd. for C_22_H_27_N_4_O_3_^+^ [M + H]^+^ 395.2078, 395.2114.

### 3.3. Synthesis of 5-Phenoxy Primaquine-Tetraoxane Conjugate ***12***

#### 3.3.1. Synthesis of 4-(4-Oxocyclohexyl)phenyl Acetate (**8**)

Acetic anhydride (141 mL, 141.9 mmol, 3.0 equiv.) was added dropwise into a solution of 4-(4-hydroxyphenyl)cyclohexanone (**8**) (9.0 g, 47.3 mmol, 1.0 equiv.) and NEt_3_ (13 mL, 94.6 mmol, 2.0 equiv.) in DCM (90 mL) at 0 °C via syringe. After complete addition, the solution was stirred at room temperature for 3 h. The mixture was washed with three solvents separately, i.e., water, sat. NaHCO_3_, and brine. Then, the organic layer were dried over anhydrous Na_2_SO_4_, concentrated to give **9** as a white solid (13.6 g, 58.42 mmol, quantitative yield). ^1^H NMR (500 MHz, CDCl_3_) δ 7.24 (d, *J* = 8.3 Hz, 2H, ArH), 7.03 (d, *J* = 8.4 Hz, 2H, ArH), 3.02 (tt, *J* = 15.0, 5.0 Hz, 1H, CH), 2.50 (dd, *J* = 10.7, 5.0 Hz, 4H, CH_2_), 2.29 (s, 3H, COCH_3_), 2.21 (d, *J* = 13.7 Hz, 2H, CH_2_), 1.91 (dd, *J* = 11.3, 7.5 Hz, 2H, CH_2_) [15].

#### 3.3.2. Synthesis of 4-(Dispiro[cyclohexane-1,3′-[1,2,4,5]tetroxane-6′,2″-tricyclo [3.3.1.13,7] decan]-4-yl)phenol (**9**)

A solution of **8** (10.0 g, 43.0 mmol, 1.0 equiv.) was dissolved in 1:1 HCO_2_H/MeCN (50 mL: 50 mL). Next, 30% H_2_O_2_ (42 mL) was slowly added into the solution at 0 °C. After complete addition, the reaction mixture was stirred at room temperature for 2 h. Then, the reaction mixture was extracted with DCM, water, sat. NaHCO_3_, and brine, separately. The combined organic layers were dried over anhydrous Na_2_SO_4_ and filtered, and the filtrate was concentrated to 50 mL. We added 2-adamantanone (6.5 g, 43.0 mmol, 1.0 equiv.) and Bi(OTf)_3_ (1.4 g, 2.2 mmol, 5 mol%) to the mixture. The reaction mixture was stirred at room temperature for 1 h. Then, the mixture was filtered through a plug of silica and concentrated. The crude product was purified by flash column chromatography (eluent: EtOAc:hexanes = 0.5:10 to 1:10) to give **9** as a white solid (413 mg, 0.99 mmol, 22%). ^1^H NMR (500 MHz, CDCl_3_) δ 7.22 (d, *J* = 8.5 Hz, 2H, ArH), 7.00 (d, *J* = 8.5 Hz, 2H, ArH), 3.24 (d, *J* = 43.5 Hz, 2H, CH), 2.61 (tt, *J* = 15.0, 5.0 Hz, 1H, CH), 2.29 (s, 3H, COCH_3_), 2.13–1.58 (m, 20H, CH/CH_2_) [15].

#### 3.3.3. Synthesis of 4-(Dispiro[cyclohexane-1,3′-[1,2,4,5]tetroxane-6′,2″-tricyclo [3.3.1.13,7] decan]-4-yl)phenol (**10**) 

A solution of **9** (410 mg, 0.96 mmol, 1.0 equiv.) was dissolved in THF (6 mL) and water (2 mL) in a round bottom flask. Next, LiOH·H_2_O (120 mg, 2.87 mmol, 3.0 equiv.) was added into the solution. The reaction mixture was then stirred at room temperature for 3 h. After completion, the mixture was neutralized with diluted HCl. Then, THF was evaporated under reduced pressure. The residue was extracted 2 times with DCM. The combined organic layers were dried over anhydrous Na_2_SO_4_, filtered, and concentrated to give **11** as a white solid (326 mg, 0.875 mmol, 92% yield). ^1^H NMR (500 MHz, CDCl_3_) δ 7.05 (d, *J* = 8.5 Hz, 2H, ArH), 6.74 (d, *J* = 8.1 Hz, 2H, ArH), 3.21 (d, *J* = 38.1 Hz, 1H, CH), 2.52 (tt, *J* = 11.8, 3.6 Hz, 1H, CH), 2.11–1.56 (m, 21H, CH/CH_2_); ^13^C NMR (126 MHz, CDCl_3_) δ 154.03, 138.20, 127.98, 115.31, 110.65, 107.73, 42.83, 37.04, 33.25, 27.15. ^1^H NMR data and ^13^C NMR data are consistent with the literature values [15].

#### 3.3.4. Synthesis of *N*^1^-(4-(4-((1*r*,3*r*,5*r*,7*r*)-Dispiro[adamantane-2,3′-[1,2,4,5]tetraoxane-6′,1″-cyclohexan]-4″-yl) phenoxy)butyl)-*N*^4^-(6-methoxy-5-phenoxyquinolin-8-yl)pentane-1,4-diamine (**12**)

1,4-Dibromobutane (48 µL, 0.402 mmol, 3.0 equiv.) was added into a solution of **10** (50 mg, 0.134 mmol, 1.0 equiv.) and dry K_2_CO_3_ (74 mg, 0.536 mmol, 4.0 equiv.) in anhydrous MeCN (2 mL) at room temperature. Then, the reaction mixture was heated to 60 °C for 6 h. Next, the reaction mixture was cooled down to room temperature and washed with water. The concentrated crude product was purified by column chromatography on silica gel using 1:4 EtOAc:hexanes to give **11** as white solid (49 mg, 0.097 mmol, 72%) [25]. To a solution of **11** (15 mg, 0.03 mmol, 1 equiv.) in anhydrous DMF (500 µL), we added K_2_CO_3_ (4 mg, 0.03 mmol, 1 equiv.) and KI (1 mg, 0.006 mmol, 0.2 equiv.), followed by a solution of **7a** (10 mg, 0.03 mmol, 1.0 equiv.) in anhydrous DMF (500 µL). The mixture was stirred at room temperature overnight. The reaction mixture was quenched with water and extracted with DCM. Then, the combined organic layers were dried over anhydrous Na_2_SO_4_, and concentrated. The crude product was purified by column chromatography (eluent: 1–3% MeOH:CH_2_Cl_2_) on silica gel to afford **12** as a yellow viscous oil (12 mg, 0.015 mmol, 51% yield). ^1^H NMR (500 MHz, CDCl_3_) δ 8.52 (ddd, *J* = 4.1, 1.6, 0.5 Hz, 1H, ArH), 8.04 (ddd, *J* = 4.1, 1.6, 0.5 Hz, 1H, ArH), 7.24–7.19 (m, 3H, ArH), 7.06 (d, *J* = 8.7 Hz, 2H, ArH), 6.95 (td, *J* = 7.3, 0.5 Hz, 1H, ArH), 6.85 (d, *J* = 7.8 Hz, 2H, ArH), 6.72 (d, *J* = 8.4 Hz, 2H, ArH), 6.54 (s, 1H, ArH), 3.90 (s, 3H, OCH_3_), 3.86–3.77 (m, 2H, OCH_2_), 3.72 (dd, *J* = 12.5, 6.3 Hz, 1H, CH), 3.22 (d, *J* = 38.2 Hz, 1H, CH), 2.98–2.81 (m, 4H, NCH_2_), 2.51 (tt, *J* = 11.6, 3.5 Hz, 1H, NH), 2.08–1.59 (m, 23H, CH/CH_2_), 1.30 (d, *J* = 6.3 Hz, 3H, CH_3_); ^13^C NMR (126 MHz, CDCl_3_) δ 159.4, 157.1, 150.5, 145.1, 142.7, 138.4, 134.0, 130.0, 129.6, 127.8, 125.1, 124.7, 122.1, 121.6, 114.9, 114.4, 110.6, 107.6, 94.8, 67.2, 57.2, 48.5, 48.3, 47.9, 42.8, 37.0, 34.4, 33.2, 29.8, 27.2, 26.7, 24.1, 20.7; HRMS (ESI+): m/z calcd. for C_47_H_59_N_3_NaO_7_ ^+^ [M + Na]^+^ 800.4245, found 800.4242.

### 3.4. Ethics Statement

Human erythrocytes were obtained from healthy volunteers aged 21–50 years following the Thai Red Cross National Blood Center protocol. All volunteers completed and signed consent forms prior to blood donation. The consent forms and blood collection protocol were approved by the BIOTEC Ethics Committee (NIRB-024-2561).

### 3.5. Parasite Culture and Blood Antimalarial Activity

*P. falciparum* strain 3D7 (wild-type drug sensitive strain) was used in this study. This parasite was maintained continuously in vitro in human O+ erythrocytes (4% hematocrit) at 37 °C under 3% CO_2_ and 90% N2 in RPMI 1640 culture media (Life Technologies Limited, Paisley, UK) containing 2 mM L-Glutamine, 25 mM HEPES (Sigma), pH 7.4, 2g/L NaHCO3, 40 mg/L gentamicin, 0.37 mM hypoxanthine, and supplemented with 5 g/L Albumax I (Life Technologies, Grand Island, NY, USA). Every 3 to 4 days, the parasite culture was synchronized with 5% sorbitol and transferred into complete medium with uninfected erythrocytes. In vitro antimalarial activity was determined by using the malaria SYBR green I-based fluorescence (MSF) method. Briefly, 0.09 mL of cultured 1% ring-stage synchronized parasites and 2% hematocrit were transferred to individual wells of a standard 96-well microtiter plate and in vitro culture continued for 48 h, with 0.01 mL of compound at different concentration in each well. The compounds were first dissolved in DMSO and diluted with RPMI medium to 1% DMSO. The final concentration of DMSO in each well was 0.1%, which caused no effect on the parasite viability. Following 48 h, SYBR Green I solution (0.02 µL of 10,000X SYBR Green I/100 µL of buffer solution consisted of 20 mM Tris 20, pH 7.5, 5 mM EDTA, 0.008% *w*/*v* saponin and 0.08% *v*/*v* Triton X-100) was then added to each well, and fluorescence signals were measured by spectrofluorometer at ex485/em 535 nm. The results were read as the concentration of each compound that exhibit 50% growth inhibition (IC50) from the dose–response curve established from the fluorescence signals at each concentration of compounds. The result of each compound was normalized with control media for the overall background subtraction as 0% and untreated parasite with 0.1% DMSO as 100% control [33,34].

### 3.6. Cytotoxicity Testing by Sulforhodamine B (SRB) Colorimetric Assay

A cytotoxicity test of selected analogs against African green monkey kidney fibroblast (Vero cells) was obtained from Bioassay Laboratory, BIOTEC, NSTDA, Thailand. They were maintained continuously in MEM/EBSS medium (Hyclone Laboratories Inc., South Logan, UT, USA), supplemented with 10% heated fetal bovine serum (GE Healthcare, PAA Laboratories GmbH, Pasching, Austria), 2.2 g/L Sodium bicarbonate (Emsure, Merck kGaA, Darmstadt, Germany), and 1% sodium pyruvate (Sigma). Cytotoxicity was determined by using the sulforhodamine B assay, and 1.9 × 104 Vero cells were incubated at 37 °C, 5% CO_2_ for 72 h. Then, the cells were fixed with 100 µL of 10% trichloroacetic acid (Sigma) at 4 °C for 45 min, washed, and dried at room temperature overnight. After this, the plate was stained with 100 µL of 0.057% (*w*/*v*) sulforhodamine B (Sigma) in 1% (*v*/*v*) acetic acid, washed with 250 µL of 1% (*v*/*v*) acetic acid, washed 4 times, and left to dry at room temperature overnight. Finally, 200 µL of 10× Tris-base was added to each well to dissolve protein-bound dye. The OD was determined at a wavelength of 510 nm. The control media was used for overall background subtraction as 0% and untreated cells as 100% control without any cytotoxic effect. The IC_50_ value of each compound was determined from the dose–response curve [35,36].

### 3.7. Inhibition of Hematin Polymerization

The ability of the 5-phenoxy primaquine analogs to inhibit hematin polymerization was investigated using a protocol modified from the one described previously [32]. Briefly, hemin chloride (30 μM; Sigma) was dispensed in a 96-well plate, followed by the addition of the compounds (1-400 μM solution in water), and the volume was adjusted to 200 μL with phosphate buffer pH 5. After it was left standing for 15 min, Tween 20 (0.5 μM; Sigma) was added. After incubation at 37 ^o^C for 1 h, the absorbance was measured at 405 nm. The assay was performed in triplicate, and results were expressed as the percentage of inhibition relative to hemozoin formation in a negative control. The IC_50_ values were obtained from the sigmoidal dose–response curves using nonlinear regression curve fitting analyses with GraphPad Prism version 3.00 software. Each IC_50_ value is the result of at least three separate experiments.

## 4. Conclusions

In summary, eight 5-phenoxy primaquine analogs bearing different substituents on the 5-phenoxy ring were successfully synthesized and characterized. Although the installation of the 5-phenoxy group increased the blood antimalarial activity slightly compared to the parent primaquine, it was found that the type of substituent on the 5-phenoxy ring did not significantly affect the activity, which could be beneficial for the modulation of pharmacokinetic properties in the drug development process. Moreover, we also successfully synthesized a novel drug hybrid (**12**) between tetraoxane and the representative 5-phenoxy primaquine **7a**. The hybrid showed substantial increase in the blood antimalarial activity with the IC_50_ of 0.38 ± 0.11 μM and relatively low toxicity against normal cell (SI = 45.61). Moreover, the study on the inhibition of hematin polymerization of the synthesized compounds revealed the additional possible mechanism of action of these analogs, which could be complementary to the usual mechanism of both primaquine and the endoperoxide. The knowledge from this study could be beneficial for the development of novel antimalarial agents with an 8-aminoquinoline core structure in the future.

## Figures and Tables

**Table 1:** Blood schizontocidal activity and cytotoxicity of 5-phenoxy primaquine analogs (**7a**–**7h**) and the tetraoxane hybrid (**12**) ^1^.

Compd.	R	IC_50_ (μM) ^2^	CC_50_ (μM) ^3^	SI ^4^
PQ	-	11.33 ± 0.79	>100	>8.8
**7a**	H	3.65 ± 0.39	37.49 ± 5.24	10.27
**7b**	OCH_3_	7.89 ± 0.50	55.62 ± 4.34	7.05
**7c**	Br	7.03 ± 1.31	42.16 ± 3.25	6.00
**7d**	Cl	8.20 ± 0.83	46.38 ± 5.68	5.66
**7e**	CN	4.62 ± 0.56	>100	>21.65
**7f**	F	4.97 ± 0.40	47.47 ± 3.23	9.55
**7g**	CF_3_	4.63 ± 0.44	22.66 ± 3.74	4.89
**7h**	CONH_2_	13.5 ± 1.57	>100	>7.41
**12**	H	0.38 ± 0.11	17.33 ± 0.36	45.61
EPT	-	ND	5.97± 0.14	-

^1^ Results are the mean ± SD obtained from three independent biological repeats; PQ = primaquine bisphosphate, EPT = ellipticine, ND = not determined; ^2^ In vitro antimalarial activity (IC_50_) against *P*. *falciparum* 3D7; ^3^ cytotoxicity (CC_50_) against African green monkey kidney fibroblast (Vero cells); ^4^ Selectivity index = CC_50_ (µM) on monkey Vero cells/IC_50_ (µM) in the blood stage.

**Table 2:** Inhibition of hemozoin-initiated hematin polymerization at pH 5.0 ^1^.

Compd.	R	IC_50_ (μM)
PQ	-	319.8 ± 12.0
**7a**	H	101.6 ± 4.1
**7b**	OCH_3_	159.5 ± 2.2
**7c**	Br	175.2 ± 4.3
**7d**	Cl	132.2 ± 5.3
**7e**	CN	285.8 ± 11.9
**7f**	F	167.1 ± 8.6
**7g**	CF_3_	109.7 ± 3.2
**7h**	CONH_2_	153.8 ± 6.4
**12**	H	66.9 ± 3.5
CQ	-	61.2 ± 1.3

^1^ Results are the mean ± SD obtained from three independent repeats; PQ = primaquine bisphosphate, CQ = chloroquine.

## Data Availability

Not applicable.

## Supplementary Materials

The following are available online. Spectroscopic data and primary data for heme polymerization assay for compounds **2**, **5**, **7a**–**7h**, **10** and **12**.

## Sample Availability

Not available.
