# Supplementary material for: 5-Phenoxy Primaquine Analogs and the Tetraoxane Hybrid as Antimalarial Agents
Source: Molecules. 2021 Jun 30;26(13):3991. doi: 10.3390/molecules26133991 (PMC8272044; doi:10.3390/molecules26133991)
Supplement: Supplementary file 1 [file molecules-26-03991-s001.zip › molecules-1271211-supplementary.pdf]

Supplementary Information

5-Phenoxy primaquine analogs and the tetraoxane hybrid as anti-malarial agents

Somruedee Jansongsaeng [1,†], Nitipol Srimongkolpithak [2,†], Jutharat Pengon [2], Sumalee Kamchonwongpaisan [2], and Tanatorn Khotavivattana [1,*]

[1] *Centre of Excellence in Natural Products Chemistry, Department of Chemistry, Faculty of Science, Chulalongkorn University, Bangkok 10330, Thailand*

[2] *National Center for Genetic Engineering and Biotechnology (BIOTEC), Pathum Thani 12120, Thailand*

**Corresponding author*

†These authors contributed equally to this work.

NMR and HRMS spectra

5-Chloro-6-methoxy-8-nitroquinoline (2)

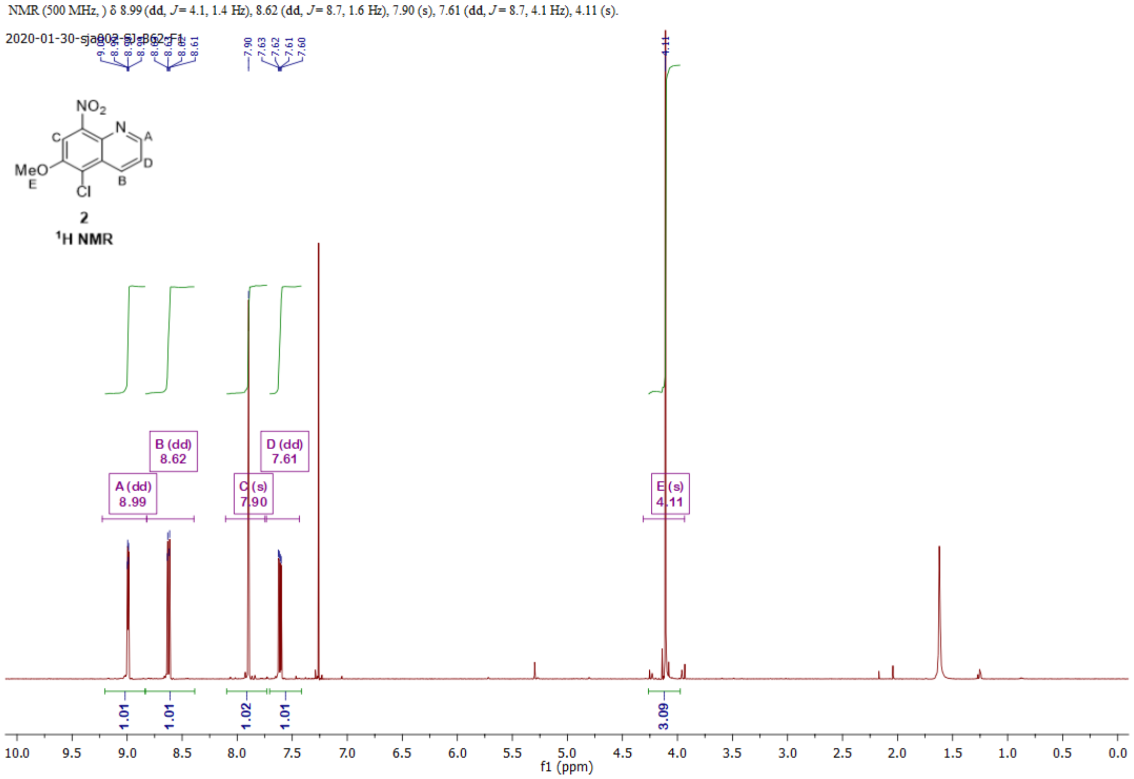

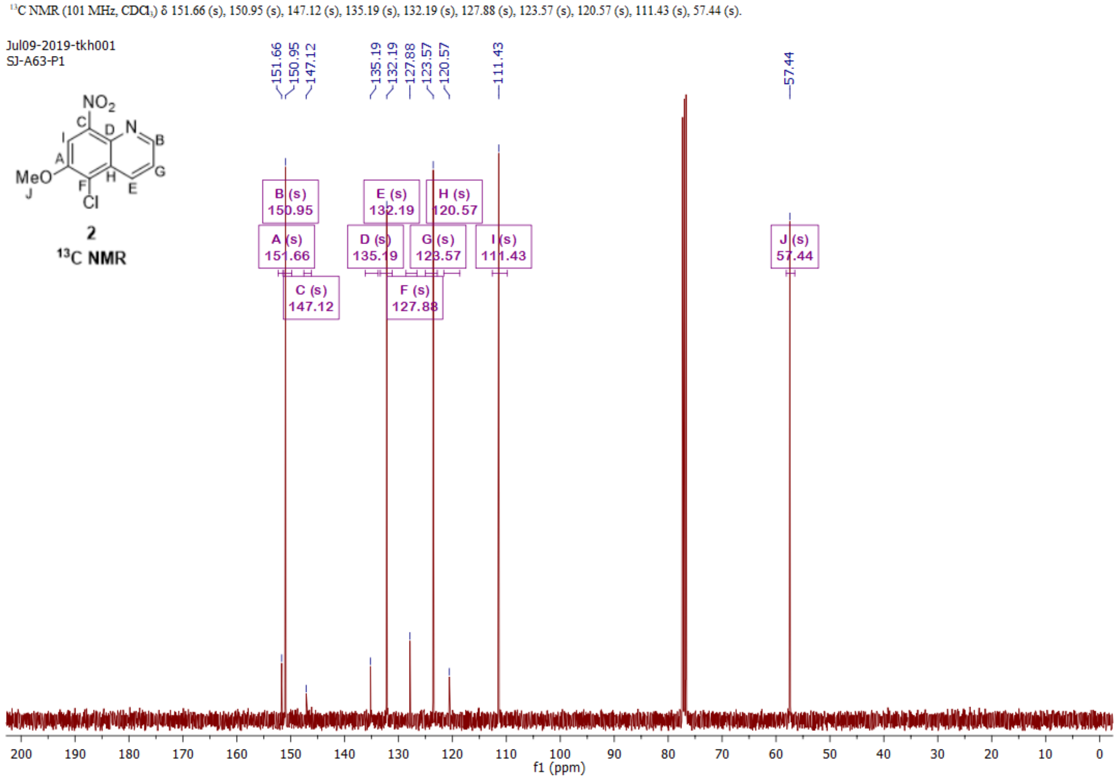

2-(4-Oxopentyl)isoindoline-1,3-dione (5)

^{1}H NMR (400 MHz, DMSO) δ 6.97 (dq, J = 6.2, 4.8 Hz, 1H), 2.67 (t, J = 6.8 Hz, 1H), 1.61 (t, J = 7.1 Hz, 1H), 1.17 (s, 1H), 0.89 (p, J = 6.9 Hz, 1H).

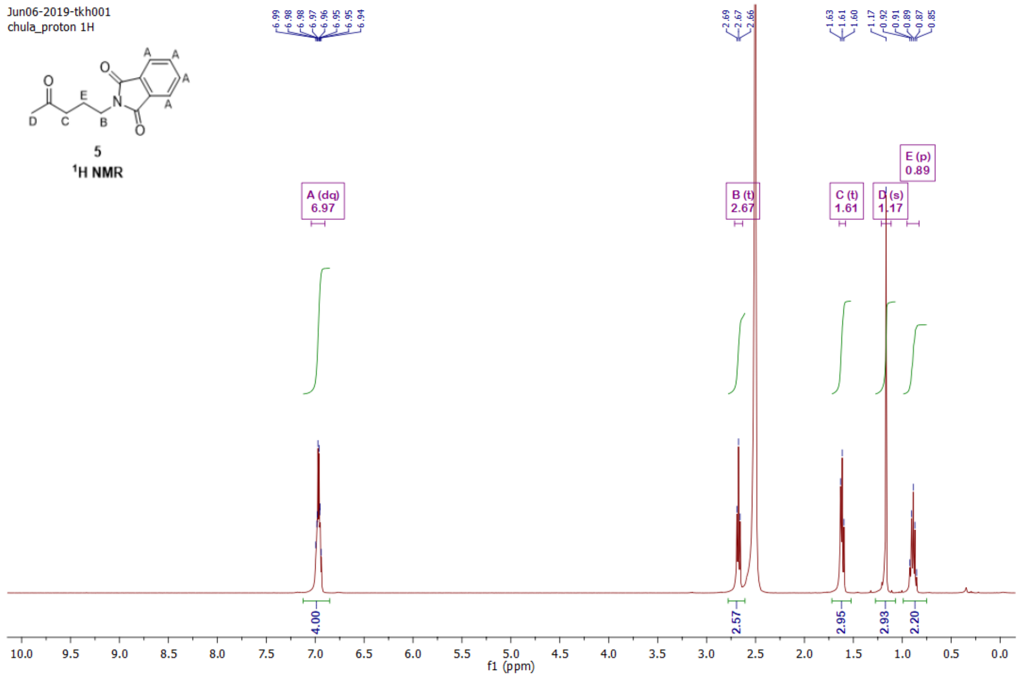

NMR (126 MHz,) δ 208.17 (s), 168.57 (s), 134.84 (s), 132.18 (s), 123.49 (s), 40.25 (s), 37.32 (s), 30.24 (s), 22.60 (s).

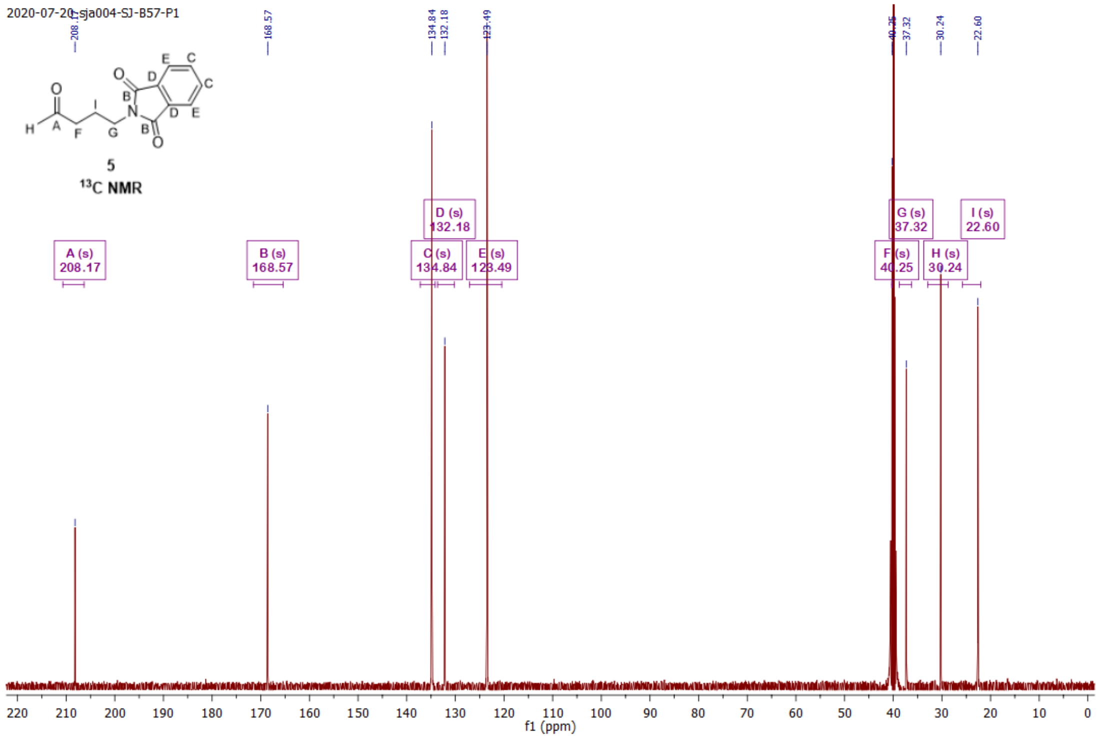

N^4-(6-Methoxy-5-phenoxyquinolin-8-yl)pentane-1,4-diamine (7a)

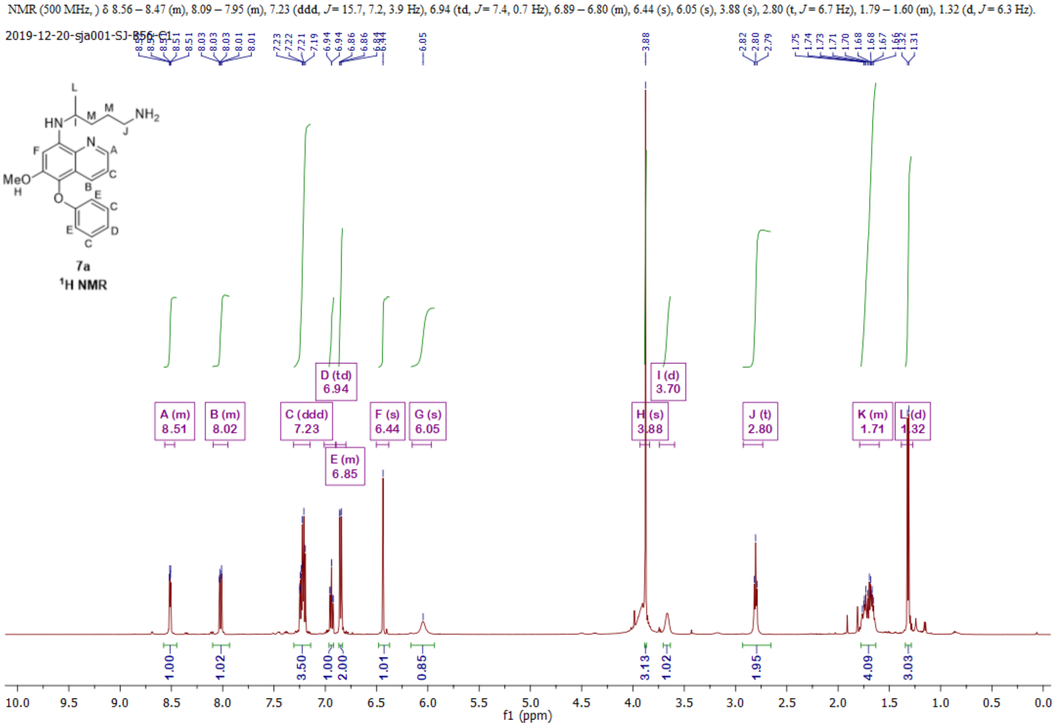

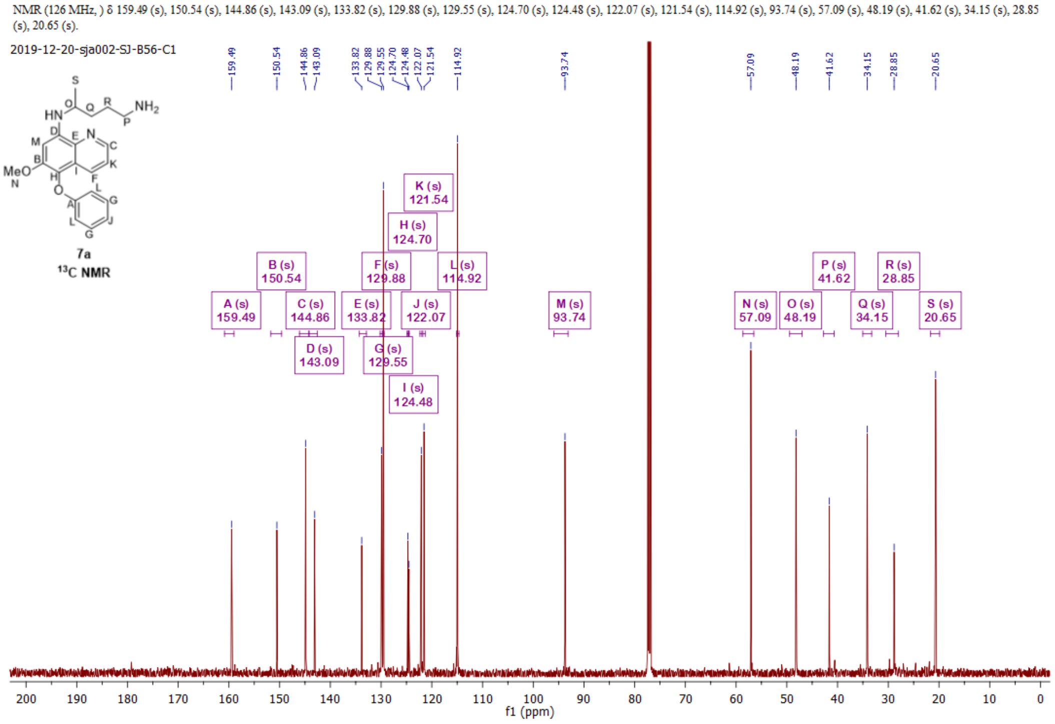

BIORESOURCES RESEARCH UNIT

High resolution report

		Acquisition Date	3/13/2020 12:24:36 PM
Analysis Name	D:\Data\customer\SJ B56 C1 H.d	Operator	Sutichai Ext: 3560
Method	NaFormate_pos.m	Instrument	micrOTOF Bruker
Sample Name	SJ B56 C1 H	Calibrate by	Sodium Formate

Acquisition Parameter

Source Type	ESI	Ion Polarity	Positive	Set Nebulizer	0.3 Bar
Focus	Not active			Set Dry Heater	180 ?C
Scan Begin	100 m/z	Set Capillary	4500 V	Set Dry Gas	4.0 l/min
Scan End	2000 m/z	Set End Plate Offset	-500 V	Set Divert Valve	Source

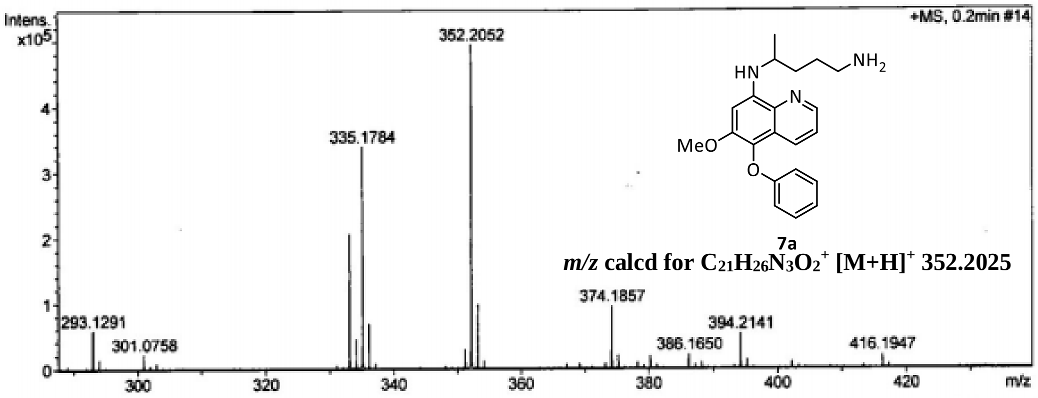

HPLC

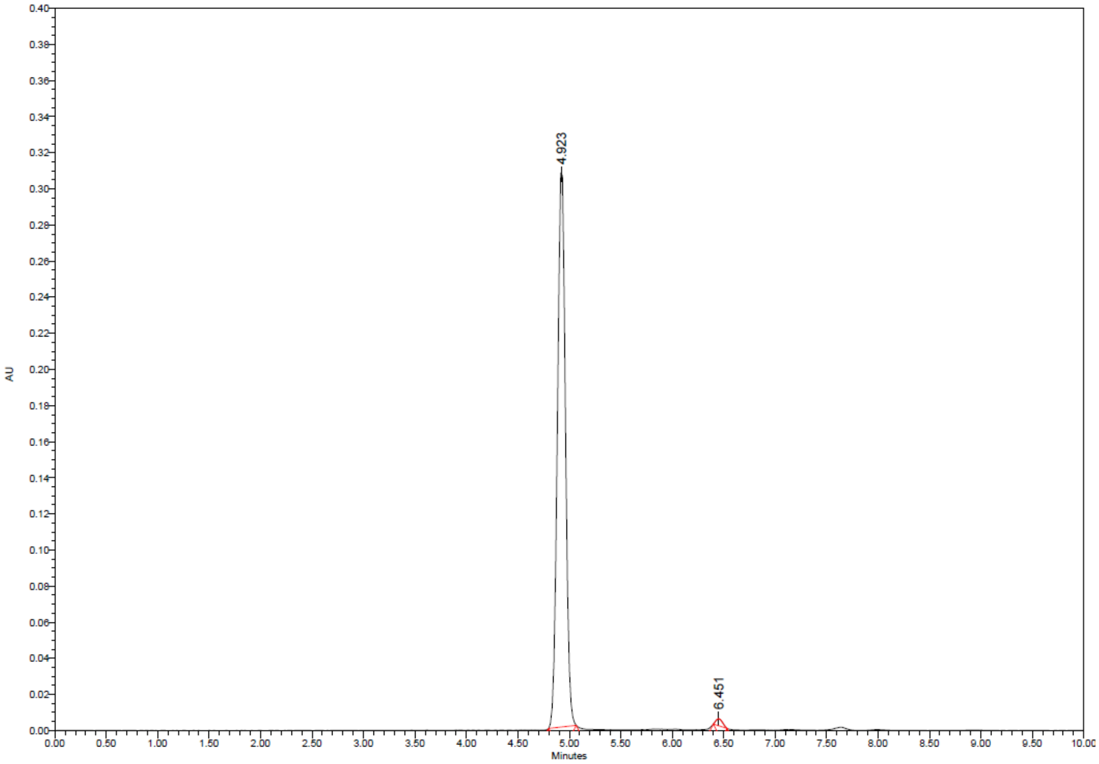

N^4-(6-Methoxy-5-(4-methoxyphenoxy)quinolin-8-yl)pentane-1,4-diamine (7b)

NMR (500 MHz,) δ 8.53 (dd, J = 4.1, 1.6 Hz), 8.05 (dd, J = 8.4, 1.5 Hz), 7.25 (dd, J = 8.5, 4.1 Hz), 6.78 (q, J = 9.3 Hz), 6.44 (s), 6.05 (s), 3.90 (s), 3.73 (s), 3.68 (dd, J = 11.9, 5.9 Hz), 2.79 (t, J = 6.8 Hz), 1.82 – 1.61 (m), 1.34 (d, J = 6.3 Hz).

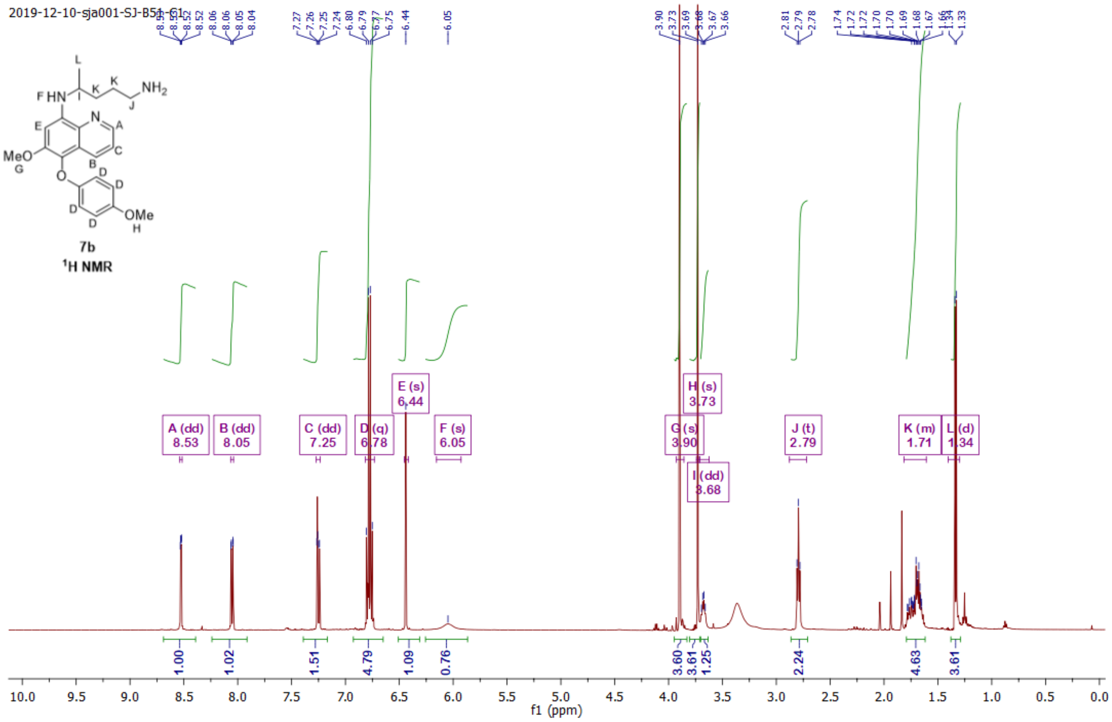

NMR (126 MHz,) δ 154.30 (s), 153.62 (s), 150.59 (s), 144.85 (s), 142.99 (s), 133.86 (s), 129.94 (s), 125.10 (s), 124.82 (s), 122.03 (s), 115.56 (s), 114.65 (s), 93.82 (s), 57.15 (s), 55.75 (s), 48.19 (s), 41.67 (s), 34.17 (s), 29.12 (s), 20.67 (s).

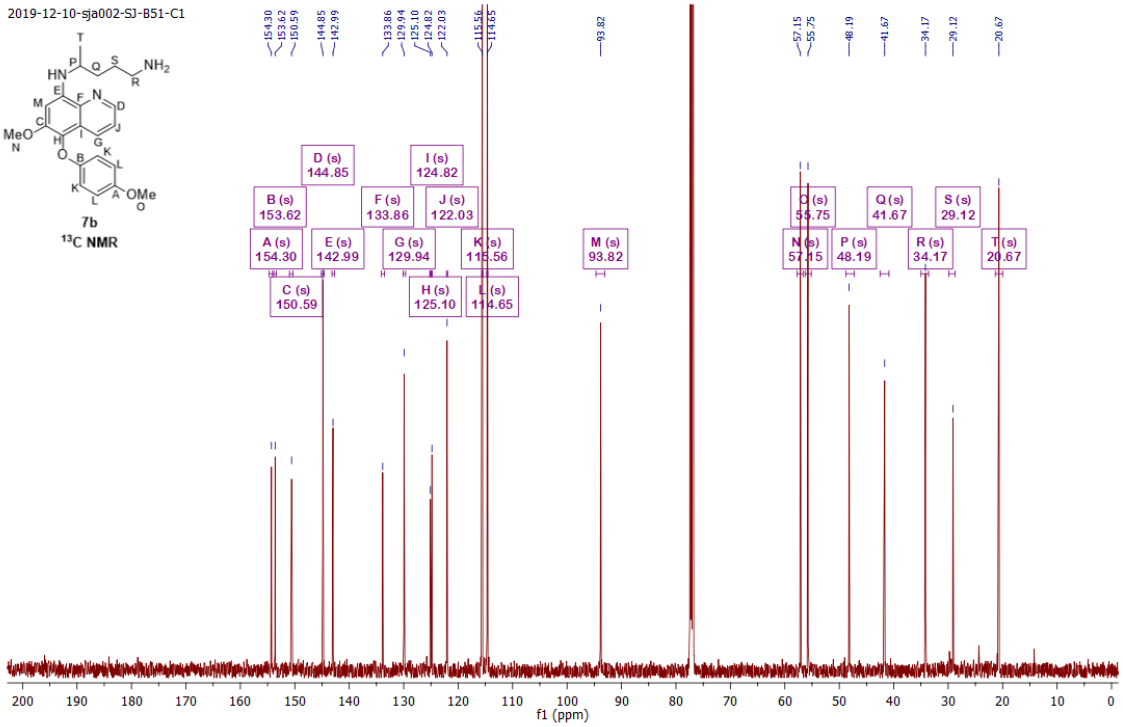

BIORESOURCES RESEARCH UNIT

High resolution report

Analysis Name	D:\Data\customer\SJ B56 C1 OMe.d
Method	NaFormate_pos.m
Sample Name	SJ B56 C1 OMe
Acquisition Date	3/13/2020 12:26:02 PM
Operator	Sutichai Ext: 3560
Instrument	micrOTOF Bruker
Calibrate by	Sodium Formate

Acquisition Parameter

Source Type	ESI	Ion Polarity	Positive	Set Nebulizer	0.3 Bar
Focus	Not active			Set Dry Heater	180 ?C
Scan Begin	100 m/z	Set Capillary	4500 V	Set Dry Gas	4.0 l/min
Scan End	2000 m/z	Set End Plate Offset	-500 V	Set Divert Valve	Source

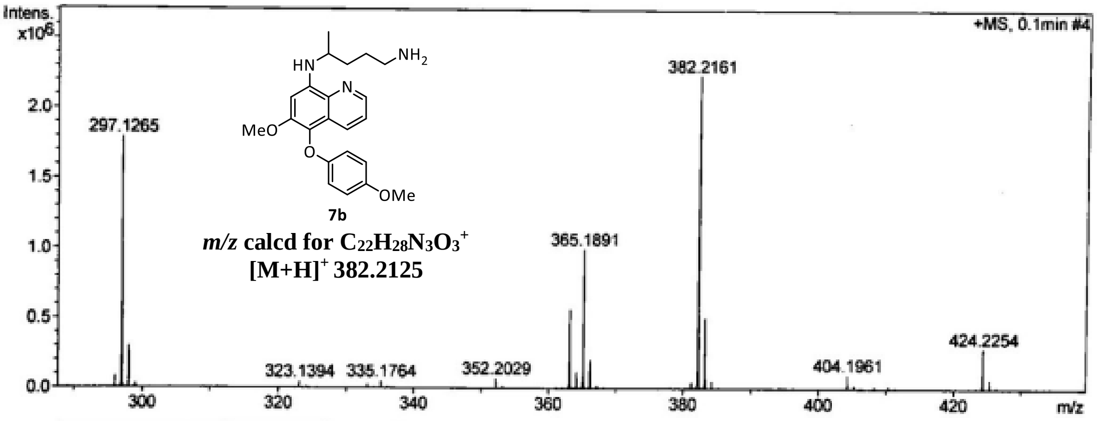

HPLC

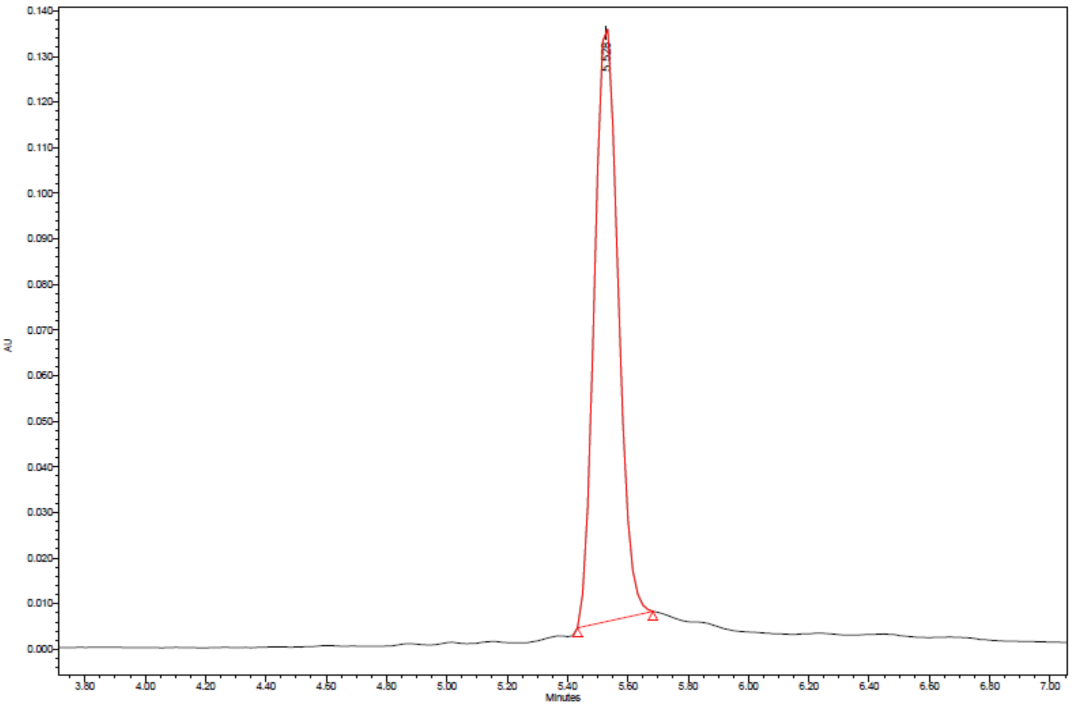

N^4-(5-(4-Bromophenoxy)-6-methoxyquinolin-8-yl)pentane-1,4-diamine (7c)

NMR (500 MHz,) δ 8.54 (dd, J = 2.7, 1.5 Hz), 7.98 (dt, J = 8.5, 1.5 Hz), 7.31 (dd, J = 9.1, 1.4 Hz), 7.27 (ddd, J = 8.5, 4.2, 1.4 Hz), 6.75 (dd, J = 9.1, 1.4 Hz), 6.43 (s), 3.89 (s), 3.68 (s), 2.78 (t, J = 6.4 Hz), 1.89 – 1.53 (m), 1.35 (d, J = 6.3 Hz).

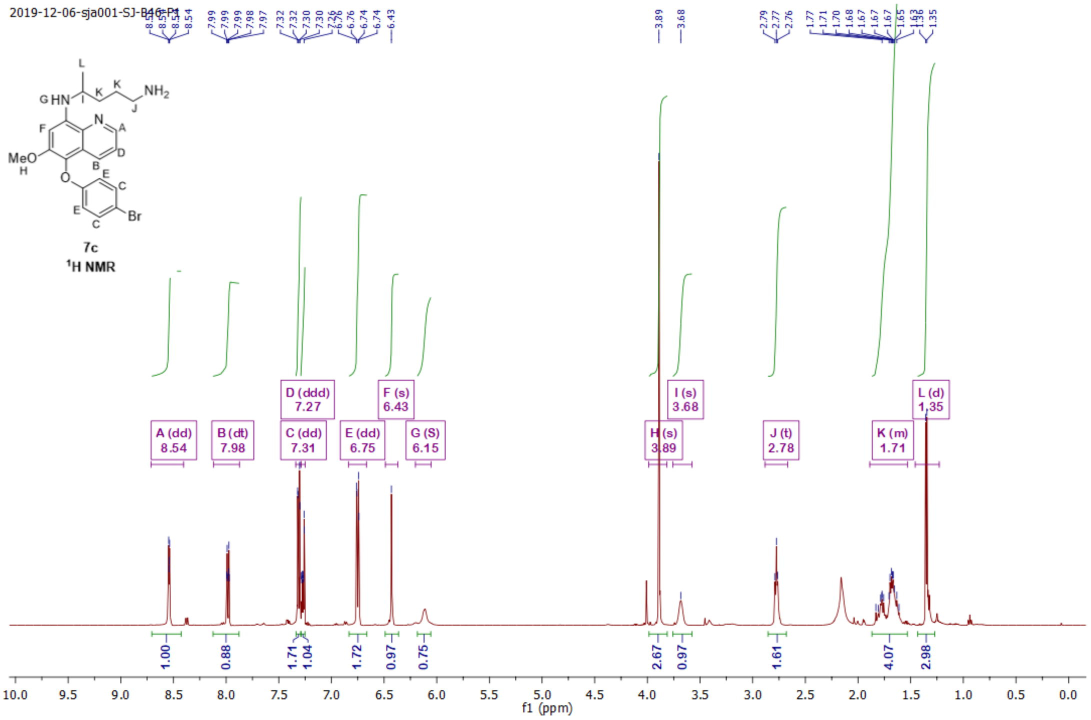

NMR (126 MHz,) δ 158.69 (s), 150.43 (s), 144.91 (s), 143.40 (s), 133.71 (s), 132.38 (s), 129.50 (s), 124.42 (s), 124.02 (s), 122.22 (s), 116.79 (s), 113.68 (s), 93.15 (s), 56.94 (s), 48.23 (s), 42.11 (s), 34.23 (s), 29.96 (s), 20.69 (s).

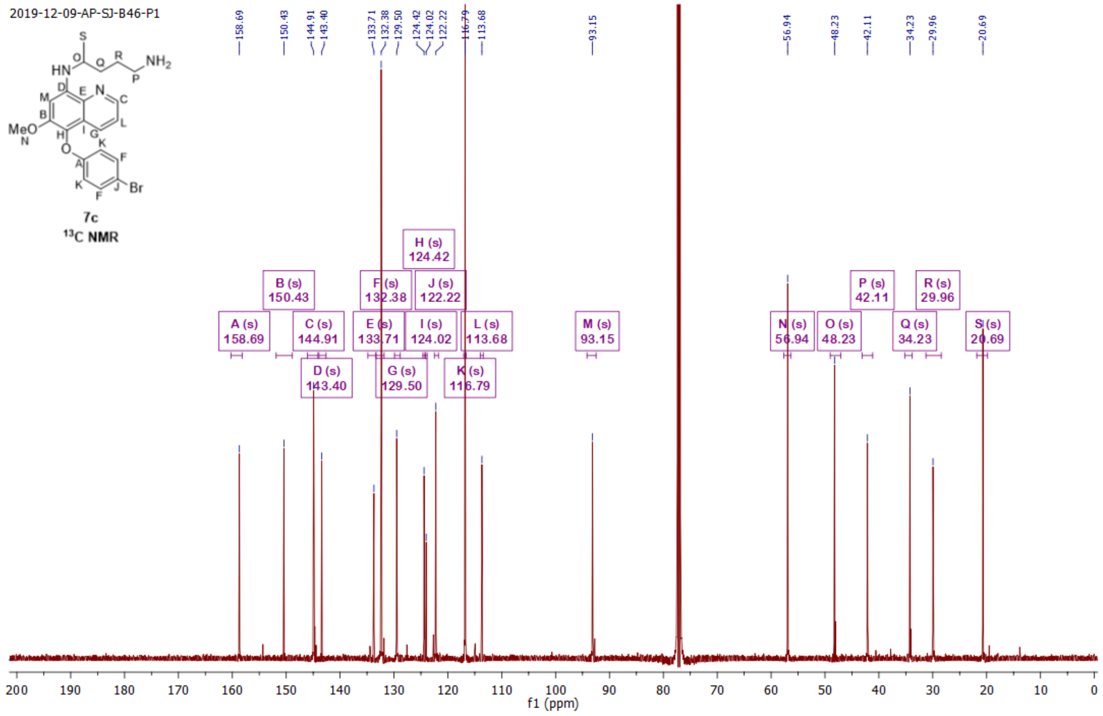

BIORESOURCES RESEARCH UNIT

High resolution report

Analysis Name	D:\Data\customer\SJ B56 P1 Br.d	Acquisition Date	3/13/2020 12:42:26 PM
Method	NaFormate_pos.m	Operator	Sutichai Ext: 3560
Sample Name	SJ B56 P1 Br	Instrument	micrOTOF Bruker
		Calibrate by	Sodium Formate

Acquisition Parameter

Source Type	ESI	Ion Polarity	Positive	Set Nebulizer	0.3 Bar
Focus	Not active			Set Dry Heater	180 ?C
Scan Begin	100 m/z	Set Capillary	4500 V	Set Dry Gas	4.0 l/min
Scan End	2000 m/z	Set End Plate Offset	-500 V	Set Divert Valve	Source

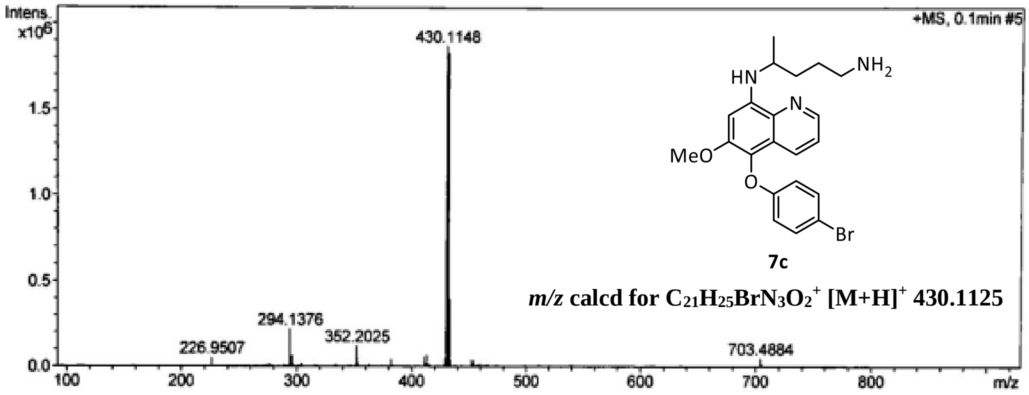

m/z calcd for $C_{21}H_{25}BrN_3O_2^+$ [M+H]$^+$ 430.1125

HPLC

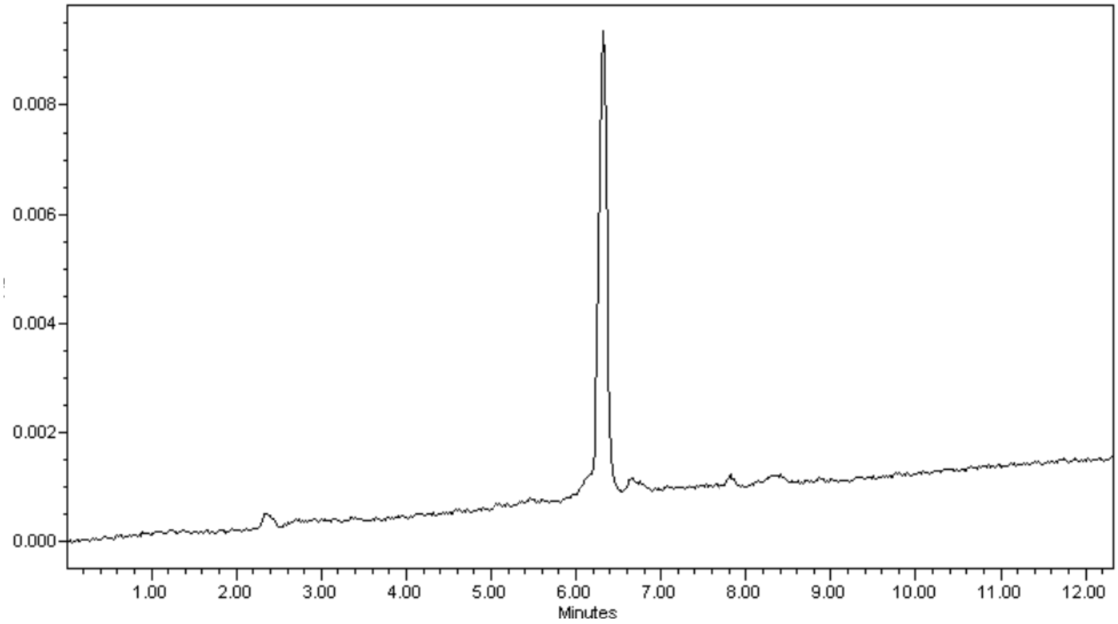

N^4-(6-Methoxy-5-(4-chlorophenoxy)quinolin-8-yl)pentane-1,4-diamine (7d)

NMR (500 MHz,) δ 8.54 (dd, J = 4.2, 1.6 Hz), 7.99 (dd, J = 8.5, 1.6 Hz), 7.30 – 7.25 (m), 7.17 (d, J = 9.0 Hz), 6.80 (d, J = 9.0 Hz), 6.43 (s), 3.89 (s), 3.69 (dd, J = 11.8, 5.8 Hz), 2.79 (t, J = 6.1 Hz), 1.80 – 1.56 (m), 1.35 (d, J = 6.3 Hz).

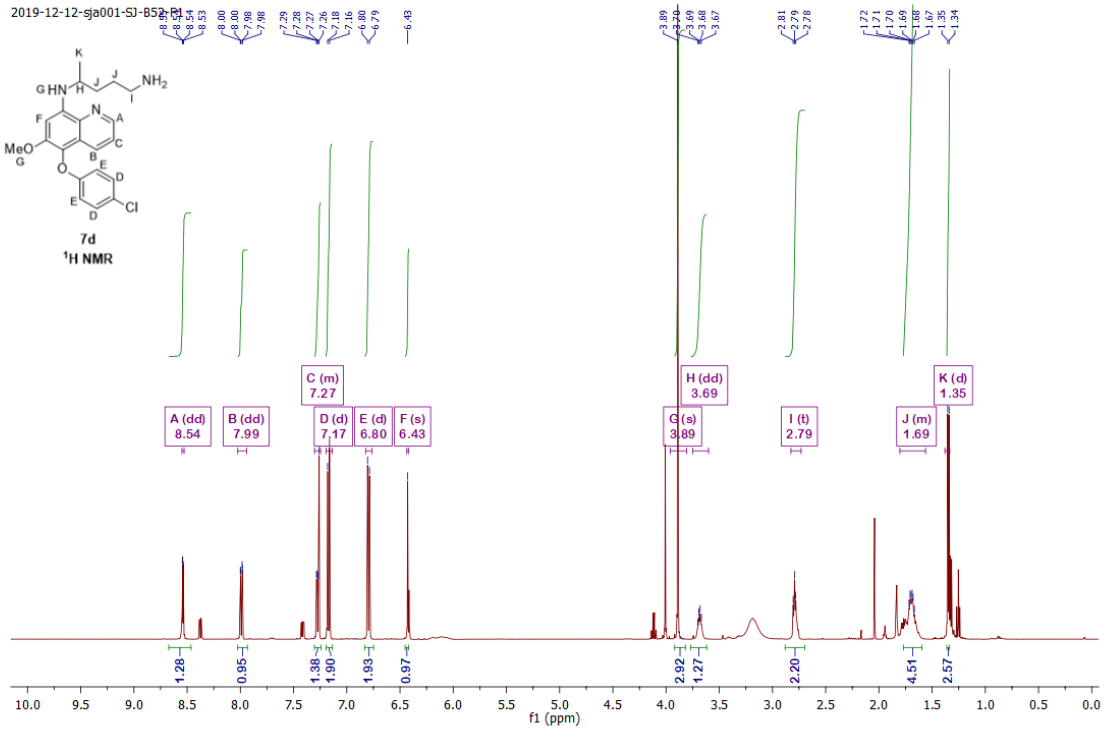

NMR (126 MHz,) δ 158.13 (s), 150.43 (s), 144.93 (s), 143.30 (s), 133.72 (s), 129.56 (s), 129.44 (s), 126.35 (s), 124.46 (s), 124.21 (s), 122.23 (s), 116.25 (s), 93.29 (s), 56.97 (s), 48.13 (s), 41.40 (s), 34.11 (s), 20.65 (s).

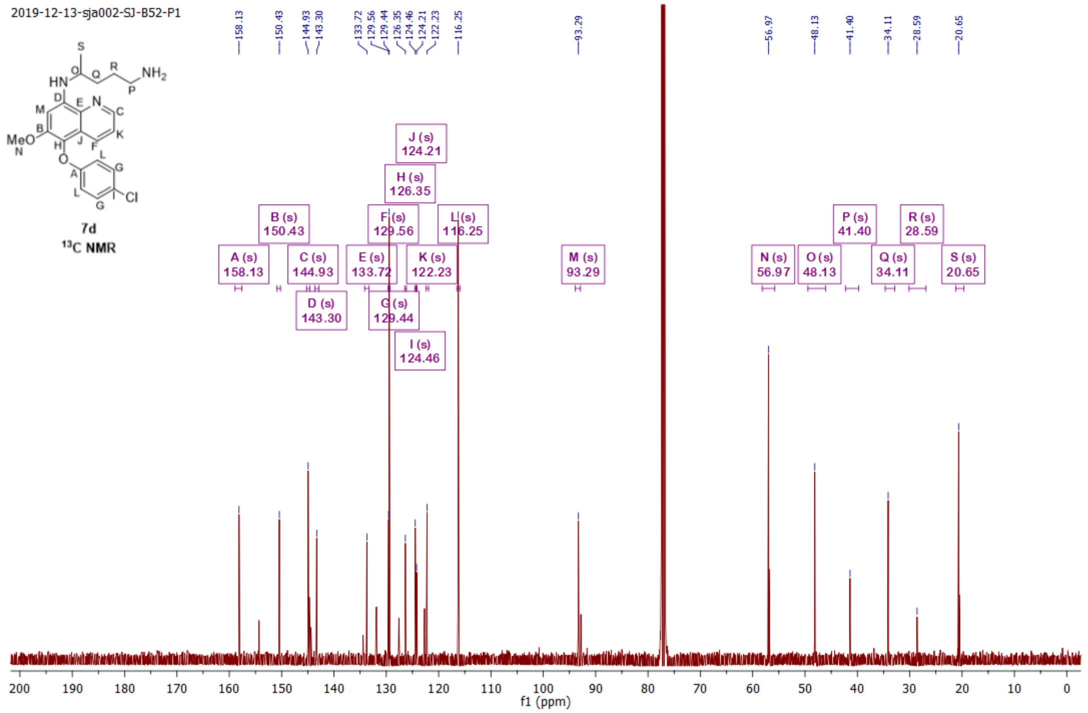

BIORESOURCES RESEARCH UNIT

High resolution report

Analysis Name	D:\Data\customer\SJ B52 P1 C1.d	Acquisition Date	3/13/2020 12:43:32 PM
Method	NaFormate_pos.m	Operator	Sutichai Ext: 3560
Sample Name	SJ B52 P1 C1	Instrument	micrOTOF Bruker
		Calibrate by	Sodium Formate

Acquisition Parameter

Source Type	ESI	Ion Polarity	Positive	Set Nebulizer	0.3 Bar
Focus	Not active			Set Dry Heater	180 ?C
Scan Begin	100 m/z	Set Capillary	4500 V	Set Dry Gas	4.0 l/min
Scan End	2000 m/z	Set End Plate Offset	-500 V	Set Divert Valve	Source

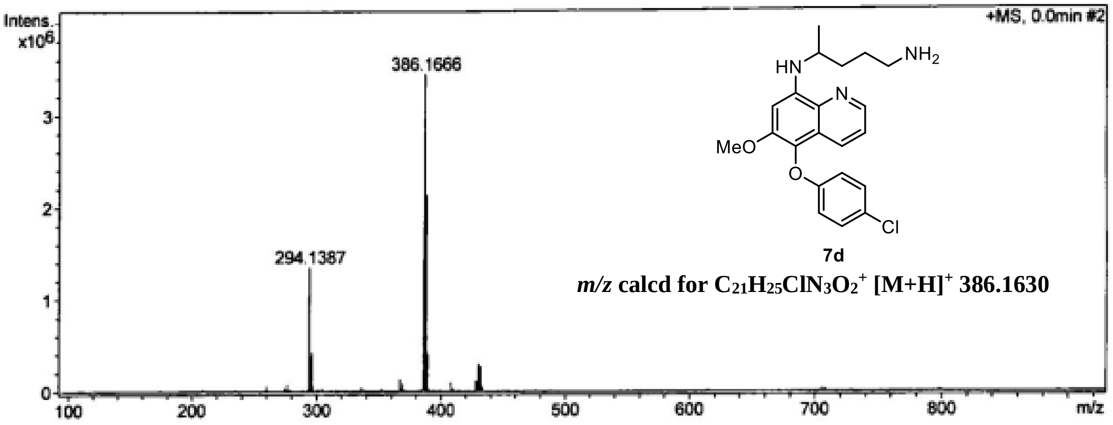

m/z calcd for $C_{21}H_{25}ClN_3O_2^+$ $[M+H]^+$ 386.1630

HPLC

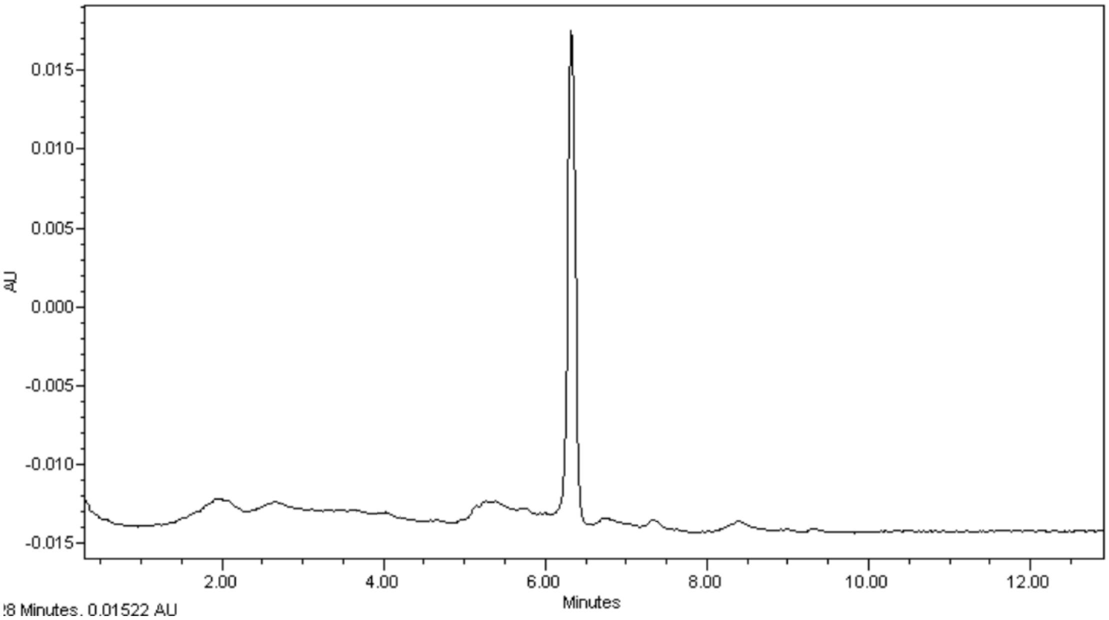

4-((8-((5-Aminopentan-2-yl)amino)-6-methoxyquinolin-5-yl)oxy)benzonitrile (7e)

NMR (500 MHz,) δ 8.56 (dd, J = 4.2, 1.4 Hz), 7.93 (dd, J = 8.5, 1.5 Hz), 7.53 (d, J = 8.7 Hz), 7.30 (dd, J = 8.5, 4.1 Hz), 6.93 (d, J = 8.7 Hz), 6.42 (s), 6.17 (s), 3.88 (s), 3.69 (s), 2.79 (t, J = 6.8 Hz), 1.82 – 1.57 (m), 1.35 (d, J = 6.3 Hz).

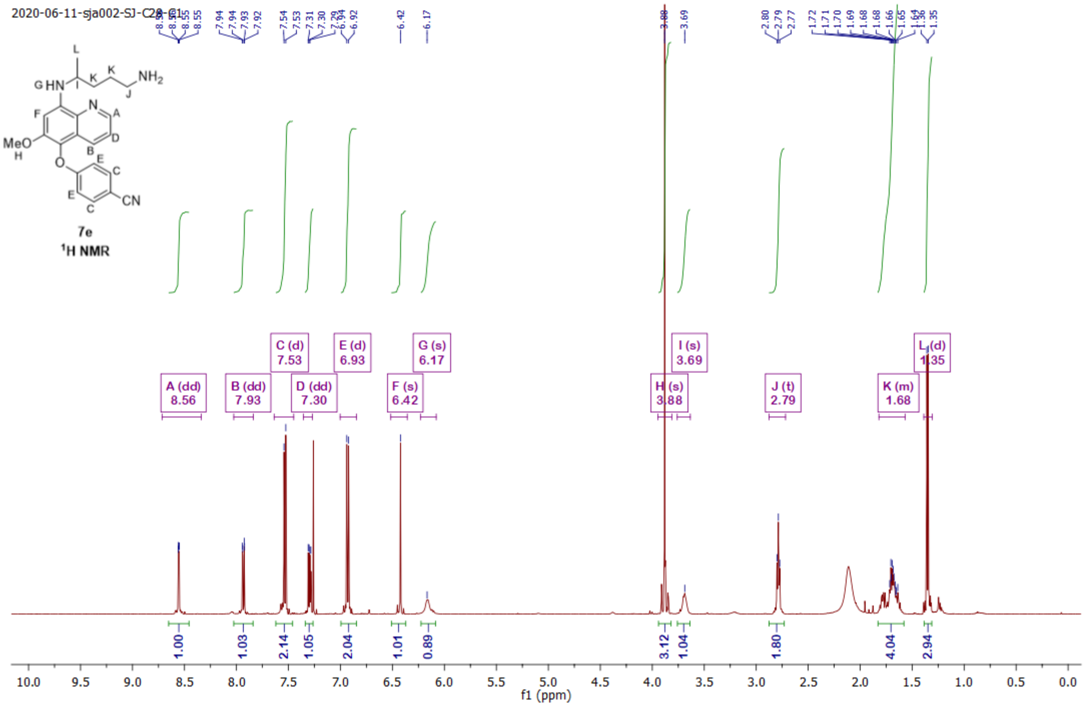

NMR (126 MHz,) δ 162.91 (s), 150.25 (s), 144.99 (s), 143.80 (s), 134.17 (s), 133.53 (s), 129.07 (s), 124.05 (s), 123.04 (s), 122.46 (s), 119.24 (s), 115.91 (s), 104.95 (s), 92.53 (s), 56.75 (s), 48.23 (s), 42.08 (s), 34.21 (s), 29.91 (s), 20.66 (s).

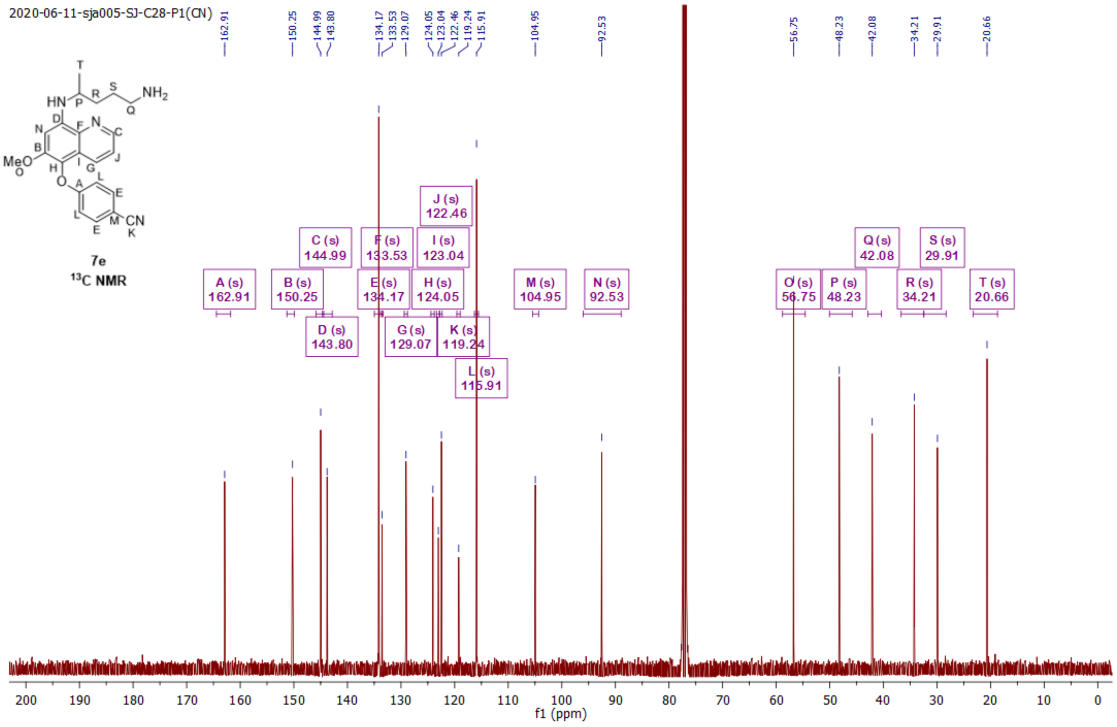

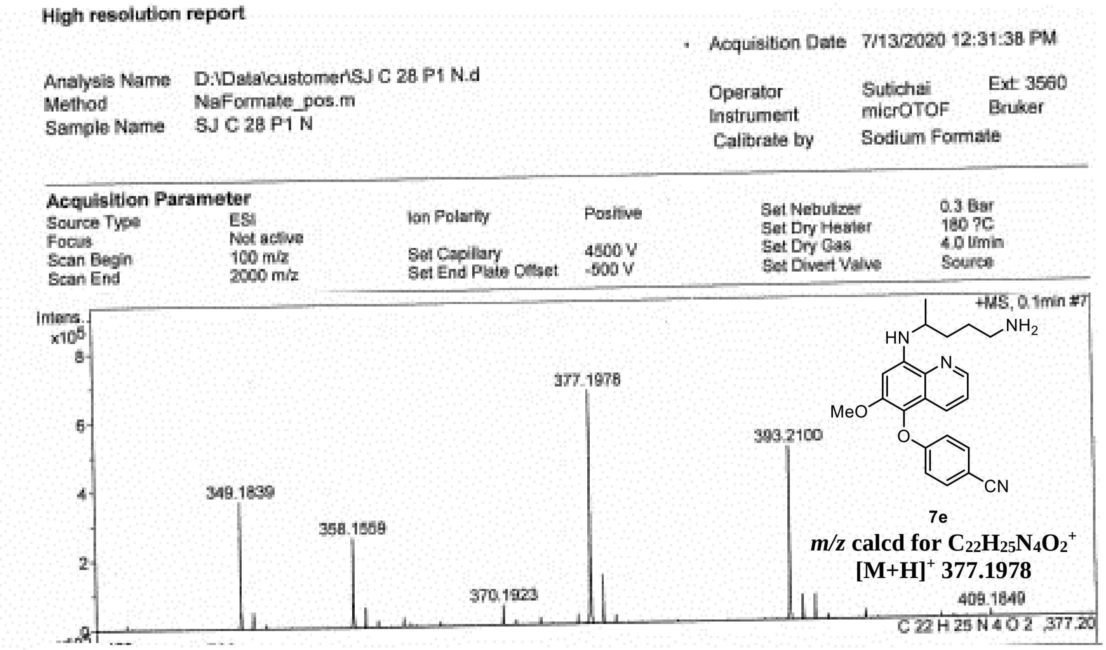
High resolution report
Acquisition Date 7/13/2020 12:31:38 PM
Analysis Name D:\Data\customer\SJ C 28 P1 N.d
Method NaFormate_pos.m
Sample Name SJ C 28 P1 N
Operator Sutichai Ext: 3560
Instrument micrOTOF Bruker
Calibrate by Sodium Formate
Acquisition Parameter
Source Type ESI
Focus Not active
Scan Begin 100 m/z
Scan End 2000 m/z
Ion Polarity Positive
Set Capillary 4500 V
Set End Plate Offset -500 V
Set Nebulizer 0.3 Bar
Set Dry Heater 180 ?C
Set Dry Gas 4.0 l/min
Set Divert Valve Source
Intens. x10^5
+MS, 0.1min #7
349.1839
358.1559
370.1923
377.1978
393.2100
409.1849
C 22 H 25 N 4 O 2 ,377.20
HN
NH2
N
MeO
O
CN
7e
m/z calcd for C22H25N4O2+
[M+H]+ 377.1978

HPLC

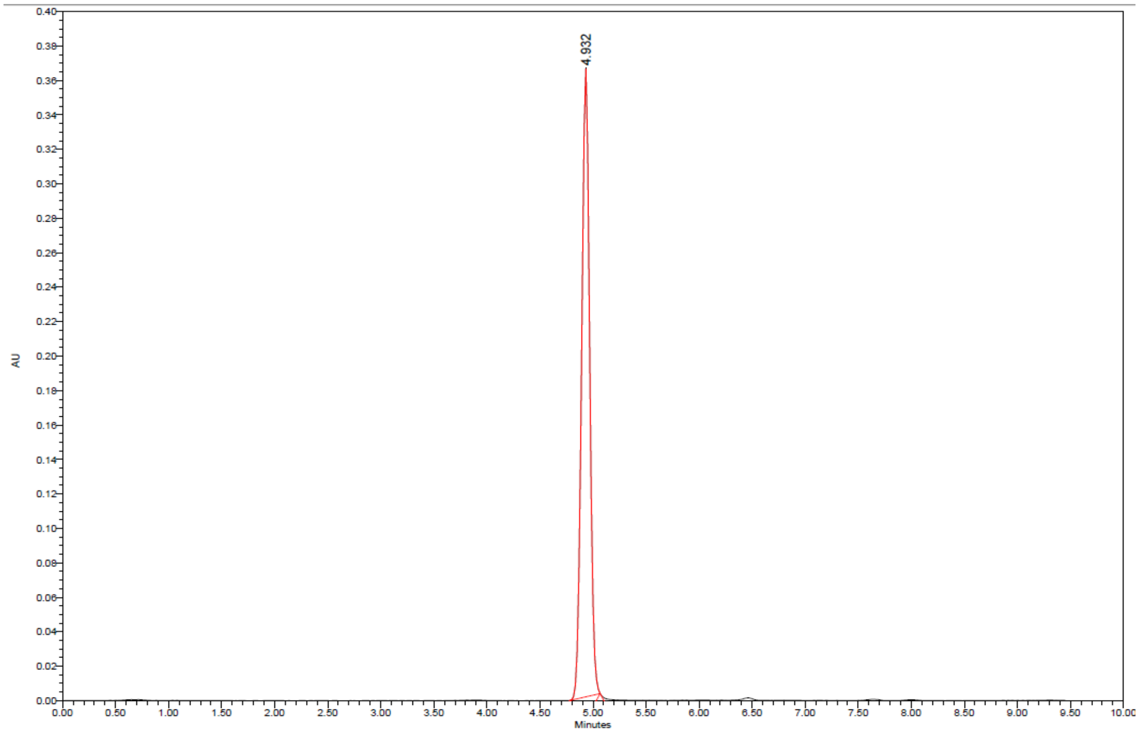
4.932
AU
Minutes

N^4-(5-(4-Fluorophenoxy)-6-methoxyquinolin-8-yl)pentane-1,4-diamine (7f)

^{1}H NMR (500 MHz, $CDCl_3$) δ 8.51 (d, J = 4.0 Hz, 1H), 8.01 (d, J = 8.4 Hz, 1H), 7.25 (dd, J = 7.7, 3.2 Hz, 1H), 6.90 (t, J = 8.6 Hz, 2H), 6.79 (dd, J = 9.0, 4.2 Hz, 2H), 6.43 (s, 1H), 6.03 (s, 1H), 3.88 (s, 3H), 3.67 (s, 1H), 2.89 (s, 2H), 1.89 – 1.62 (m, 4H), 1.30 (d, J = 6.0 Hz, 3H).

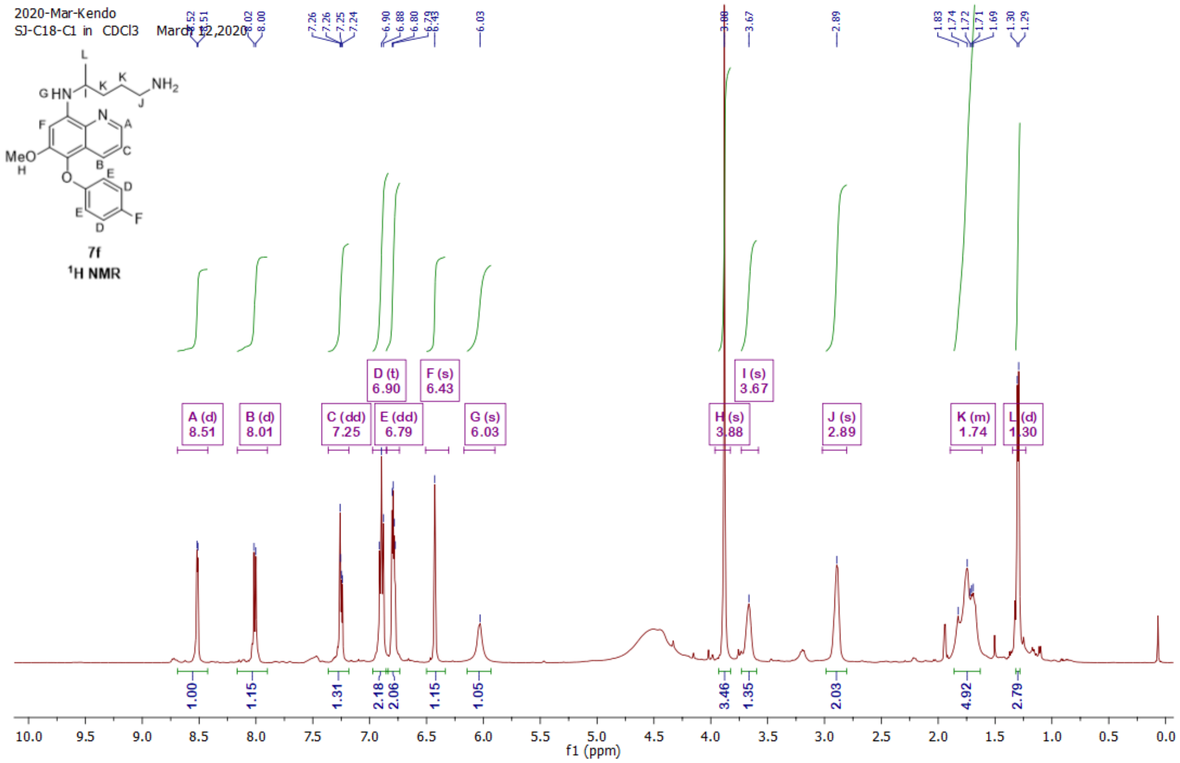

NMR (126 MHz,) δ 158.71 (s), 156.09 (d, J = 177.0 Hz), 150.61 (s), 144.71 (s), 142.71 (s), 124.96 (s), 124.67 (s), 122.15 (s), 116.28 (d, J = 7.9 Hz), 115.90 (d, J = 31.5 Hz), 115.84 (s), 94.26 (s), 57.09 (s), 48.09 (s), 40.02 (s), 33.75 (s), 24.58 (s), 20.50 (s).

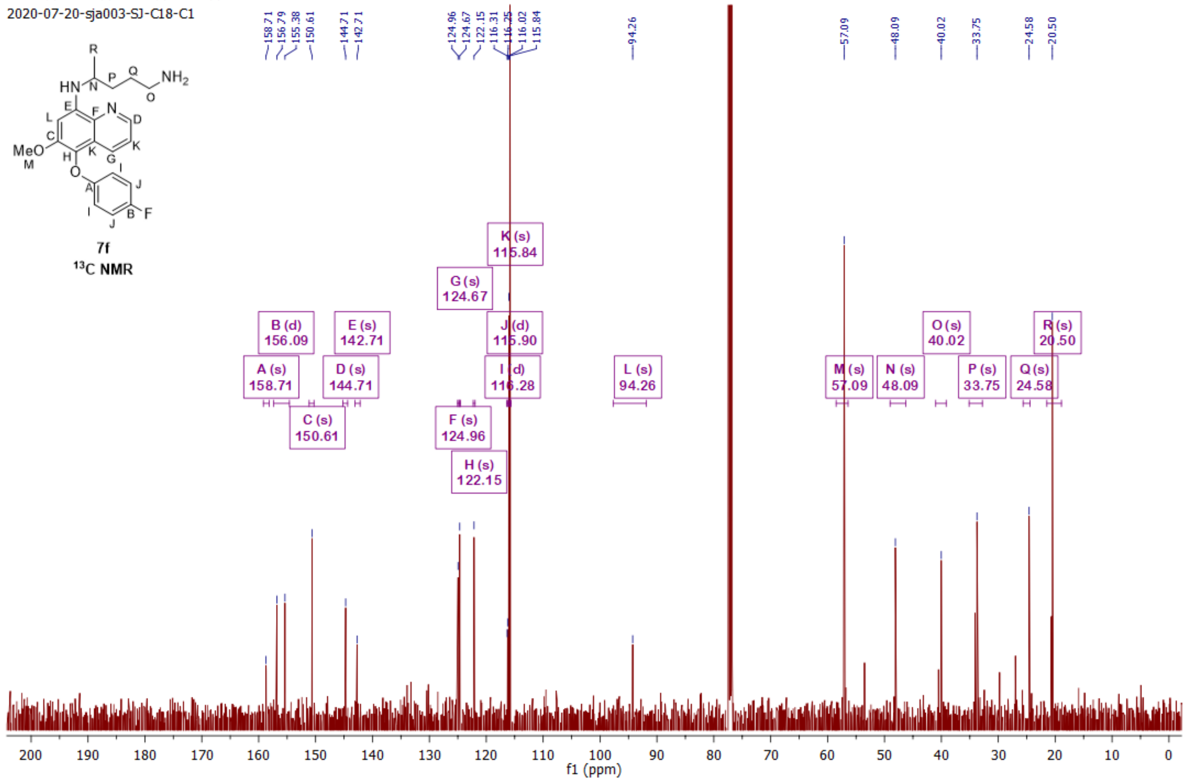

^{19}F NMR (471 MHz, $CDCl_3$) δ -123.31 (s).

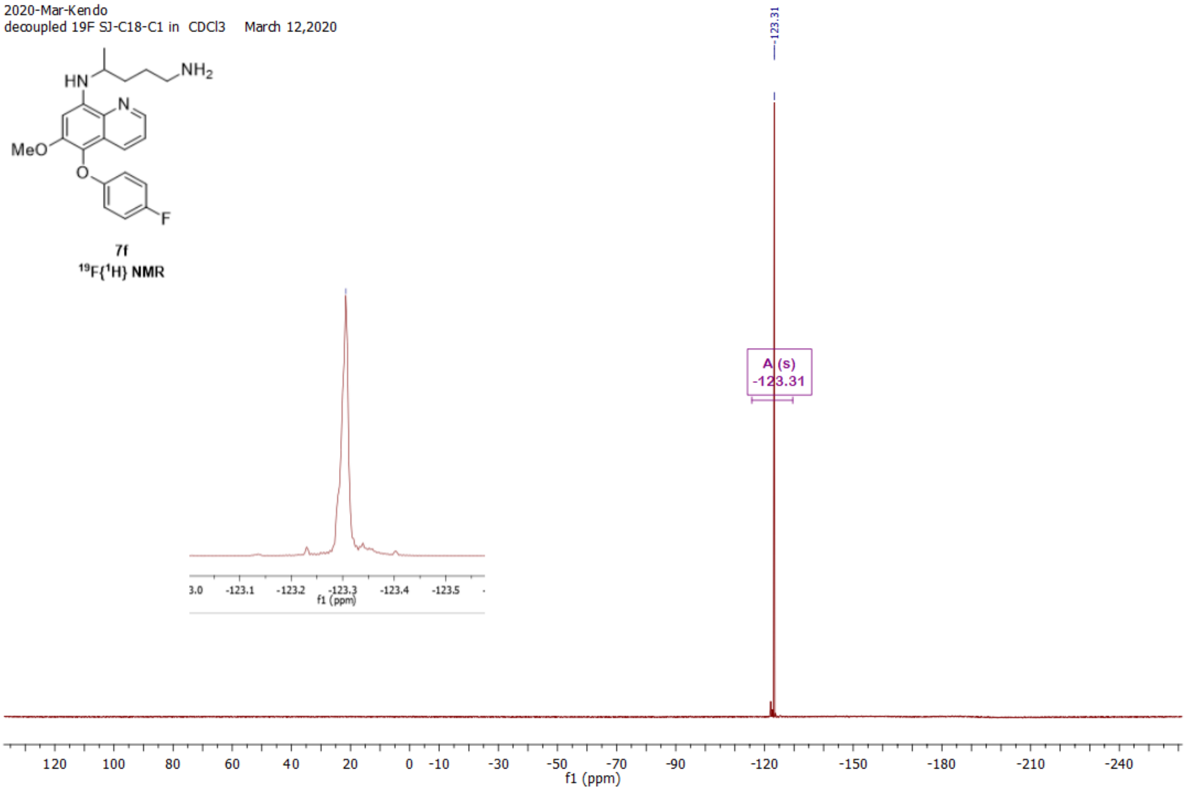

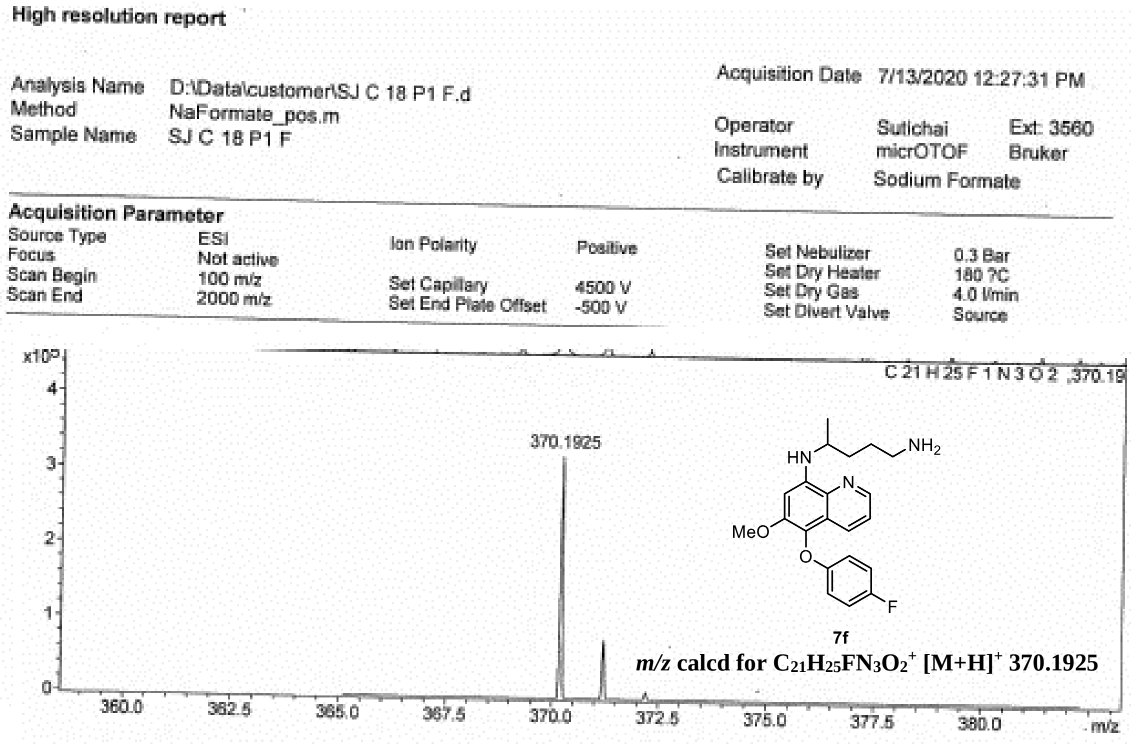

HPLC

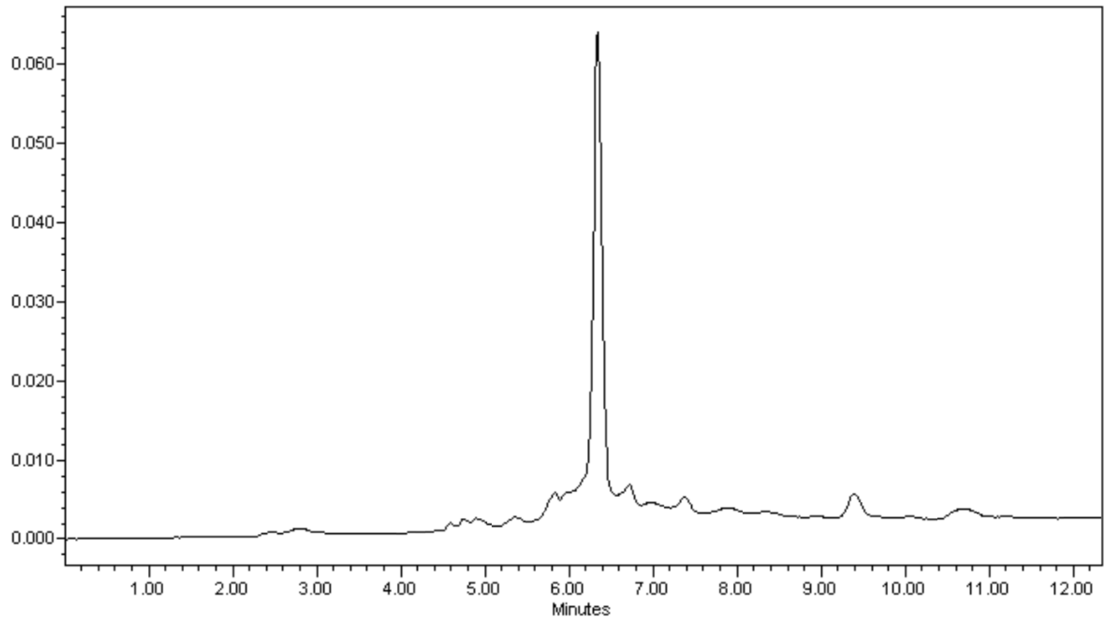

0.060
0.050
0.040
0.030
0.020
0.010
0.000
1.00
2.00
3.00
4.00
5.00
6.00
7.00
8.00
9.00
10.00
11.00
12.00
Minutes

N^4-(5-(4-Fluorophenoxy)-6-methoxyquinolin-8-yl)pentane-1,4-diamine (7g)

NMR (500 MHz,) δ 8.68 – 8.53 (m), 8.00 (ddd, J = 8.5, 3.5, 1.7 Hz), 7.61 (d, J = 8.0 Hz), 7.41 (ddd, J = 8.3, 4.1, 1.5 Hz), 7.01 (d, J = 8.3 Hz), 6.71 (dd, J = 16.1, 14.9 Hz), 3.91 (dd, J = 4.4, 1.7 Hz), 3.29(m), 1.94 (t, J = 6.2 Hz), 2.98 - 2.79 (m) 1.86 – 1.68 (m), 1.35 (d, J = 6.3 Hz).

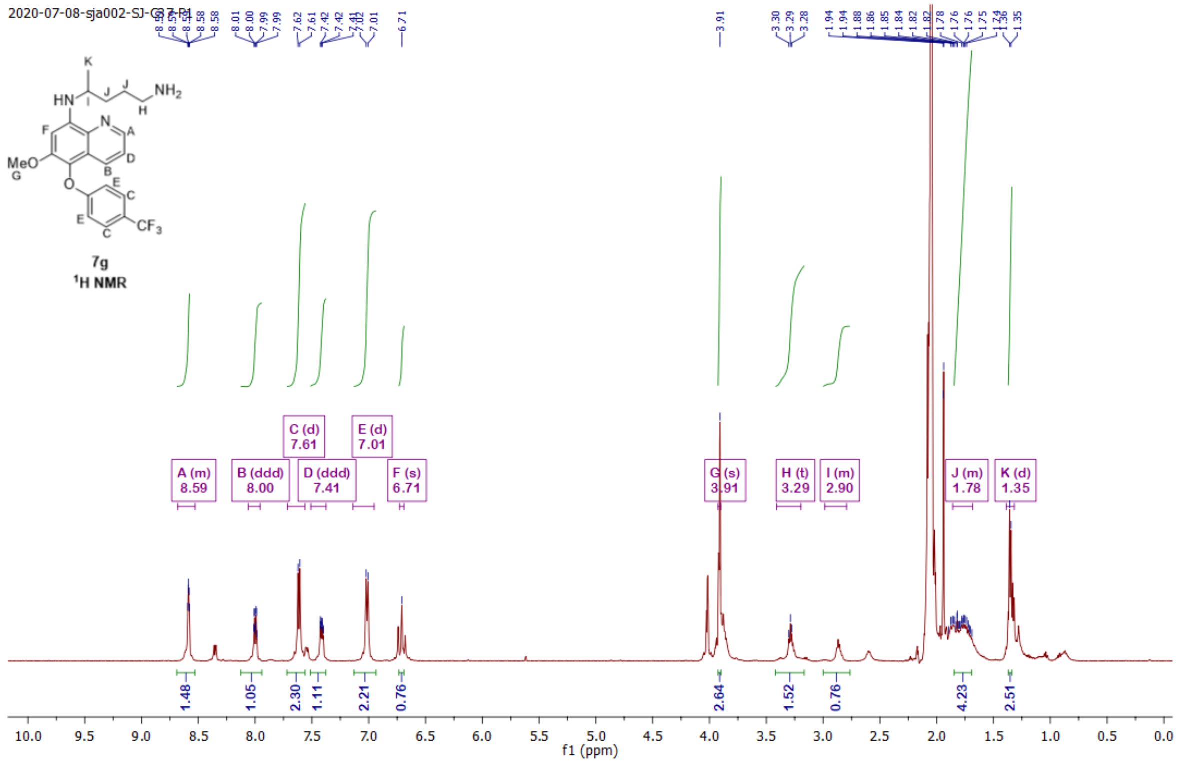

NMR (126 MHz,) δ 150.72 (s), 144.80 (s), 144.58 (s), 144.02 (s), 133.39 (s), 131.04 (s), 128.68 (q, J = 4.1 Hz), 126.94 (q, J = 65.1 Hz), 124.02 (s), 122.87 (d, J = 7.7 Hz), 122.69 (q, J = 265.0 Hz), 122.48 (s), 115.31 (s), 92.73 (s), 56.14 (s), 50.51 (s), 47.88 (s), 41.21 (s), 34.57 (s), 20.05 (s).

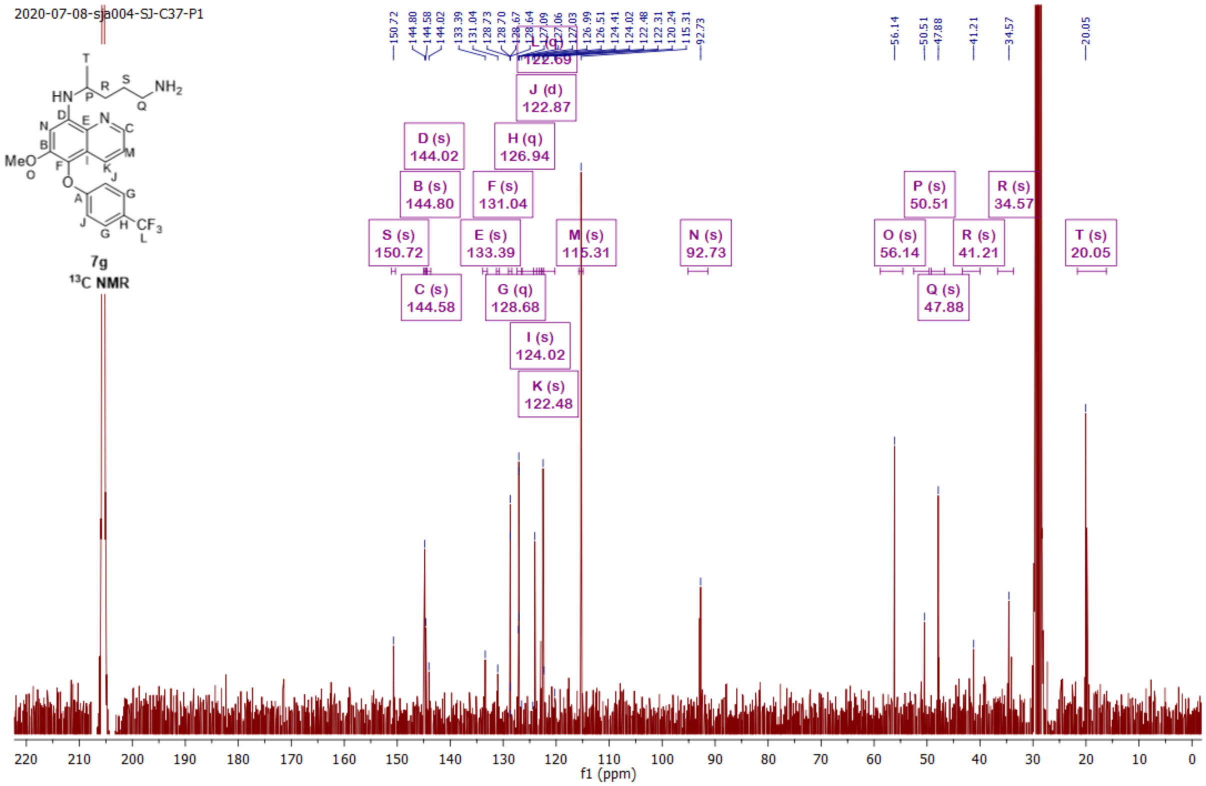

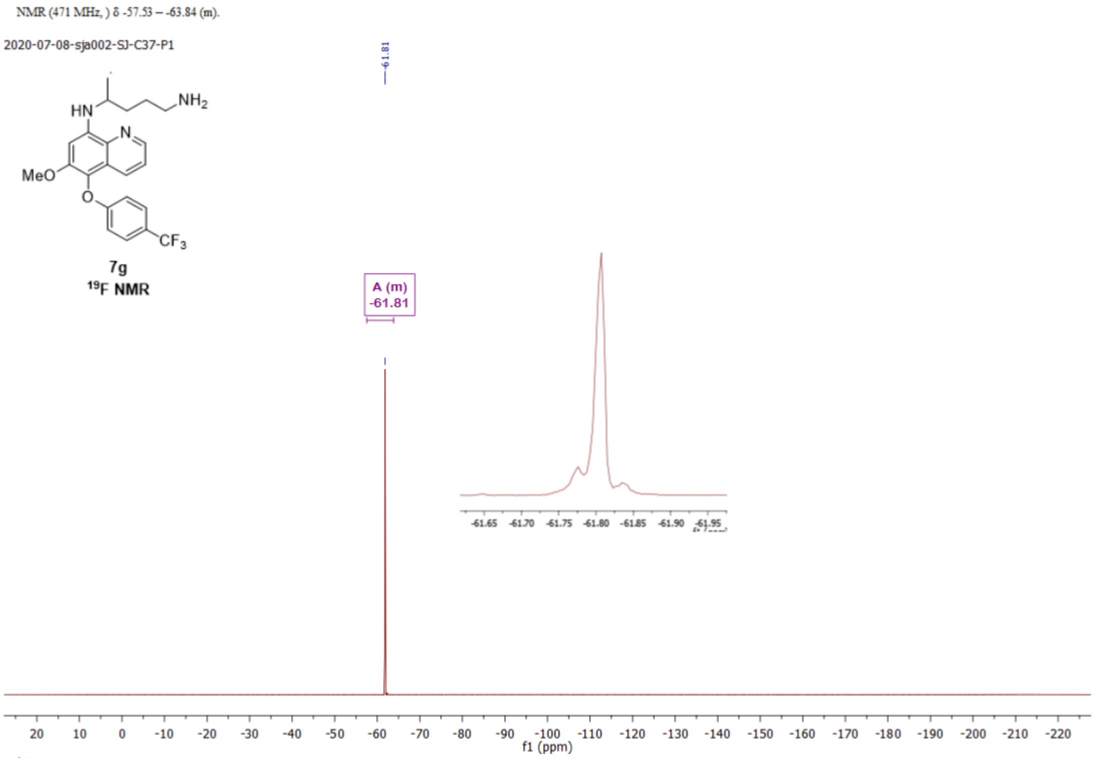

High resolution report

		Acquisition Date	17/11/2563 13:24:20	
Analysis Name	\\10.222.72.169\Data\customer\SJC37P1CF3.d			
Method	NaFormate_pos.m	Operator	Sutichai	Ext: 3560
Sample Name	SJC37P1CF3	Instrument	micrOTOF	Bruker
		Calibrate by	Sodium Formate	

Acquisition Parameter

Source Type	ESI	Ion Polarity	Positive	Set Nebulizer	1.5 Bar
Focus	Not active			Set Dry Heater	200 °C
Scan Begin	50 m/z	Set Capillary	4500 V	Set Dry Gas	8.0 l/min
Scan End	3000 m/z	Set End Plate Offset	-500 V	Set Divert Valve	Source

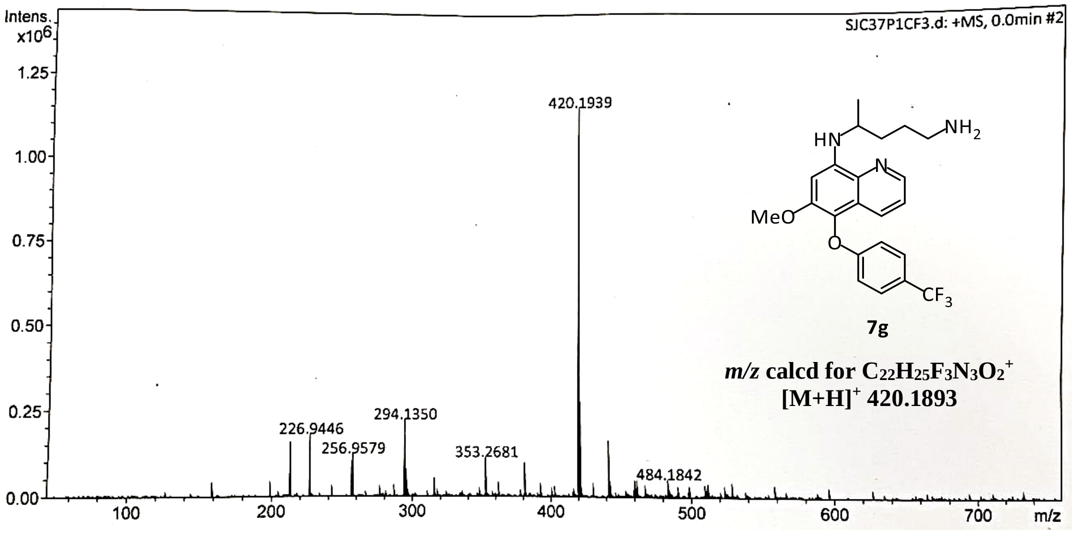

HPLC

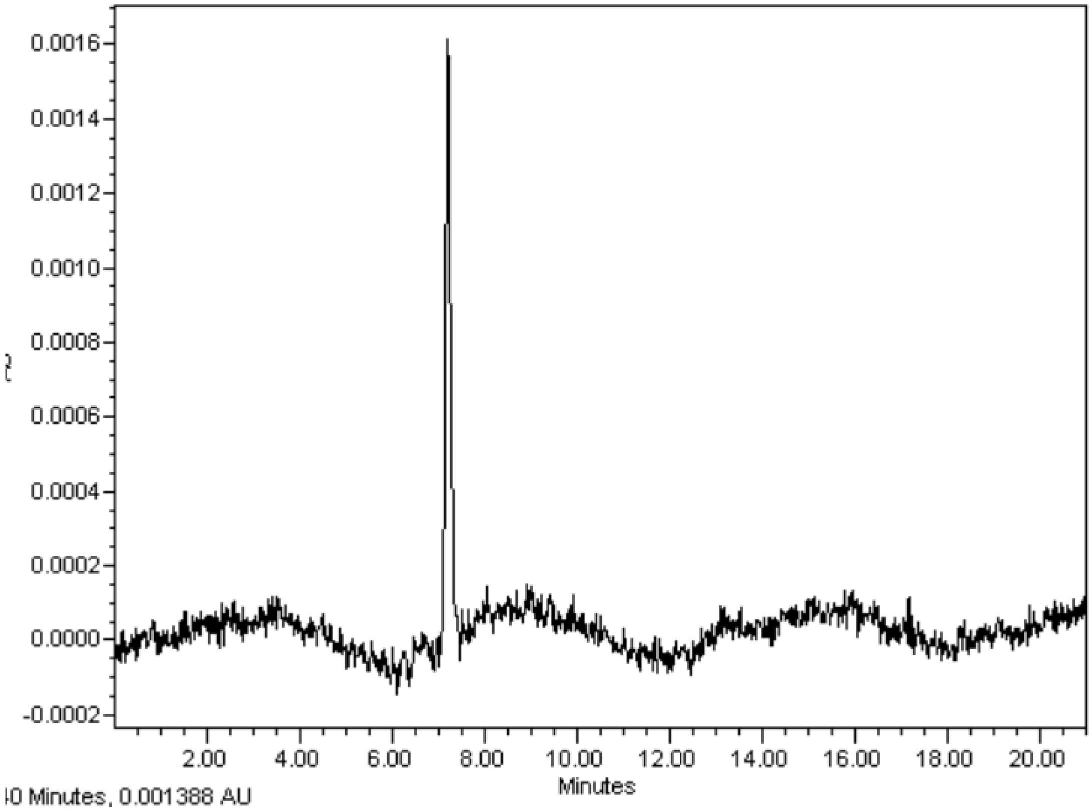
0.0016
0.0014
0.0012
0.0010
0.0008
0.0006
0.0004
0.0002
0.0000
-0.0002
2.00
4.00
6.00
8.00
10.00
12.00
14.00
16.00
18.00
20.00
Minutes
0 Minutes, 0.001388 AU

4-((8-((5-Aminopentan-2-yl)amino)-6-methoxyquinolin-5-yl)oxy)benzamide (7h)

NMR (500 MHz,) δ 8.51 (dd, J = 4.1, 1.4 Hz), 8.16 (dd, J = 5.7, 3.4 Hz), 7.96 (dd, J = 11.1, 4.1 Hz), 7.77 (s), 7.76 (s), 7.32 (dd, J = 8.4, 4.1 Hz), 6.81 (dd, J = 8.3, 1.4 Hz), 6.58 (d, J = 4.1 Hz), 3.84 (s), 3.82 – 3.77 (m), 2.95 (t, J = 7.2 Hz), 1.76 (t, J = 10.6 Hz), 1.31 (t, J = 5.5 Hz).

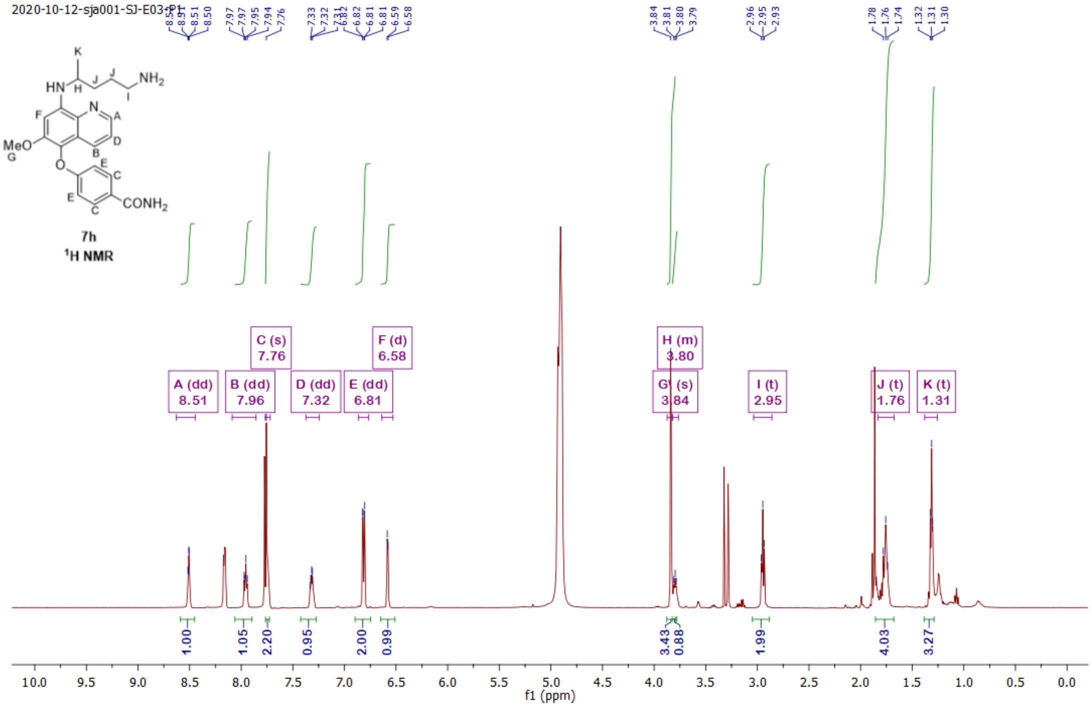

NMR (126 MHz,) δ 170.51 (s), 162.49 (s), 150.44 (s), 144.76 (s), 143.46 (s), 133.37 (s), 131.65 (s), 129.34 (s), 128.97 (s), 126.72 (s), 125.56 (s), 124.22 (s), 123.76 (s), 122.07 (s), 114.36 (s), 93.29 (s), 55.84 (s), 39.61 (s), 33.30 (s), 19.53 (s).

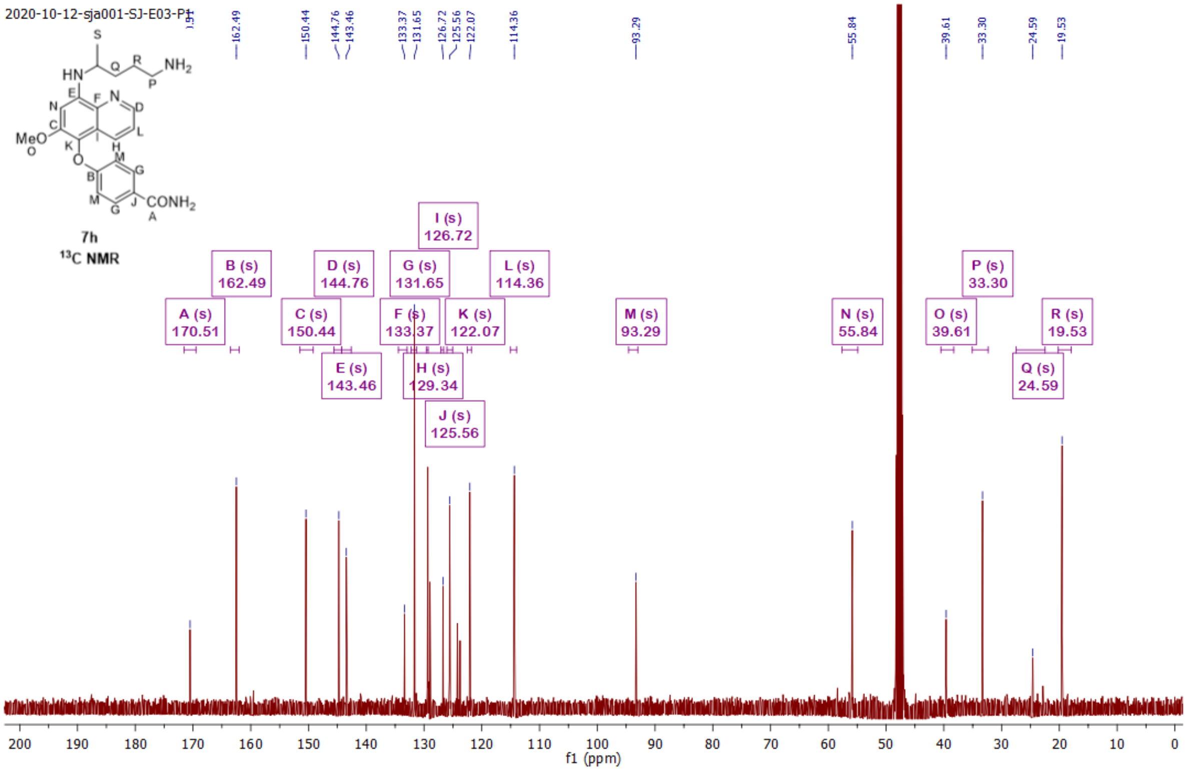

Mass Spectrum List Report

Analysis Info

Analysis Name	D:\Data\Data Service\201214\SJ-E03-P1_RC4_01_5064.d	Acquisition Date	12/14/2020 6:45:27 PM
Method	nv_pos_5min_profile_190214.m	Operator	CU.
Sample Name	SJ-E03-P1	Instrument / Ser#	micrOTOF-Q II 10335
Comment			

Acquisition Parameter

Source Type	ESI	Ion Polarity	Positive	Set Nebulizer	3.0 Bar
Focus	Not active	Set Capillary	4000 V	Set Dry Heater	200 ℃
Scan Begin	100 m/z	Set End Plate Offset	-500 V	Set Dry Gas	8.0 l/min
Scan End	1500 m/z	Set Collision Cell RF	250.0 Vpp	Set Divert Valve	Waste

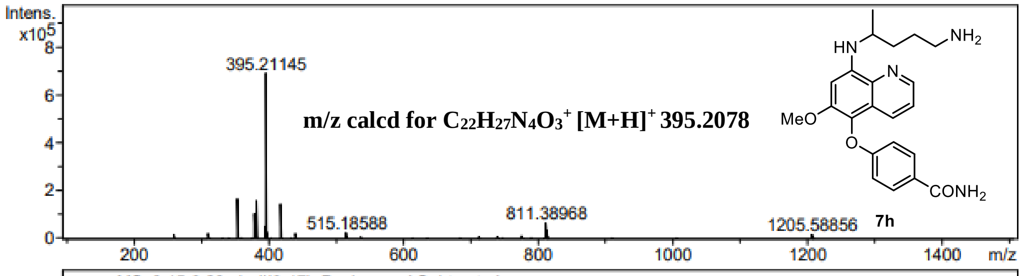

m/z calcd for $C_{22}H_{27}N_4O_3^+$ [M+H]$^+$ 395.2078

7h

+MS, 0.15-0.29min #(9-17), Background Subtracted

4-(Dispiro[cyclohexane-1,3'-[1,2,4,5]tetroxane-6',2''-tricyclo[3.3.1.13,7]decan]-4-yl)phenol (10)

NMR (500 MHz,) δ 7.05 (d, J = 8.5 Hz), 6.74 (d, J = 8.1 Hz), 3.21 (d, J = 38.1 Hz), 2.52 (tt, J = 11.8, 3.6 Hz), 2.11 – 1.56 (m).

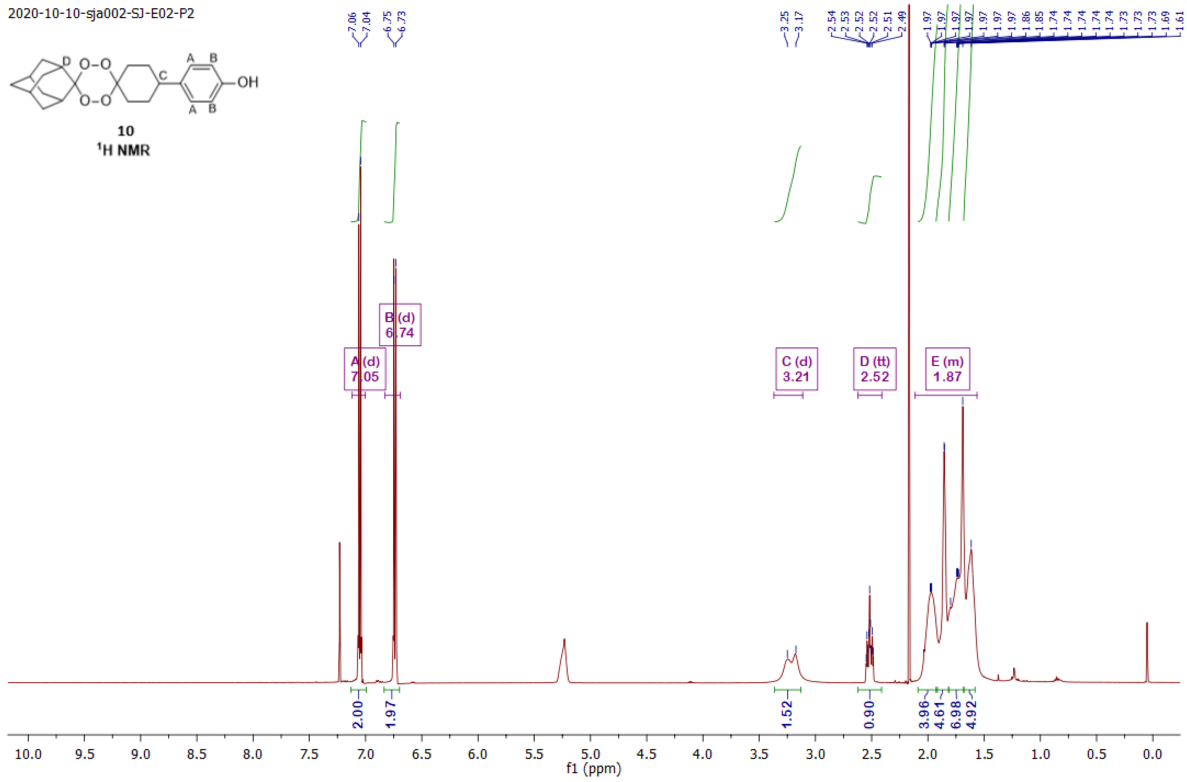

NMR (126 MHz,) δ 154.03 (s), 138.20 (s), 127.98 (s), 115.31 (s), 110.65 (s), 107.73 (s), 42.83 (s), 37.04 (s), 33.25 (s), 27.15 (s).

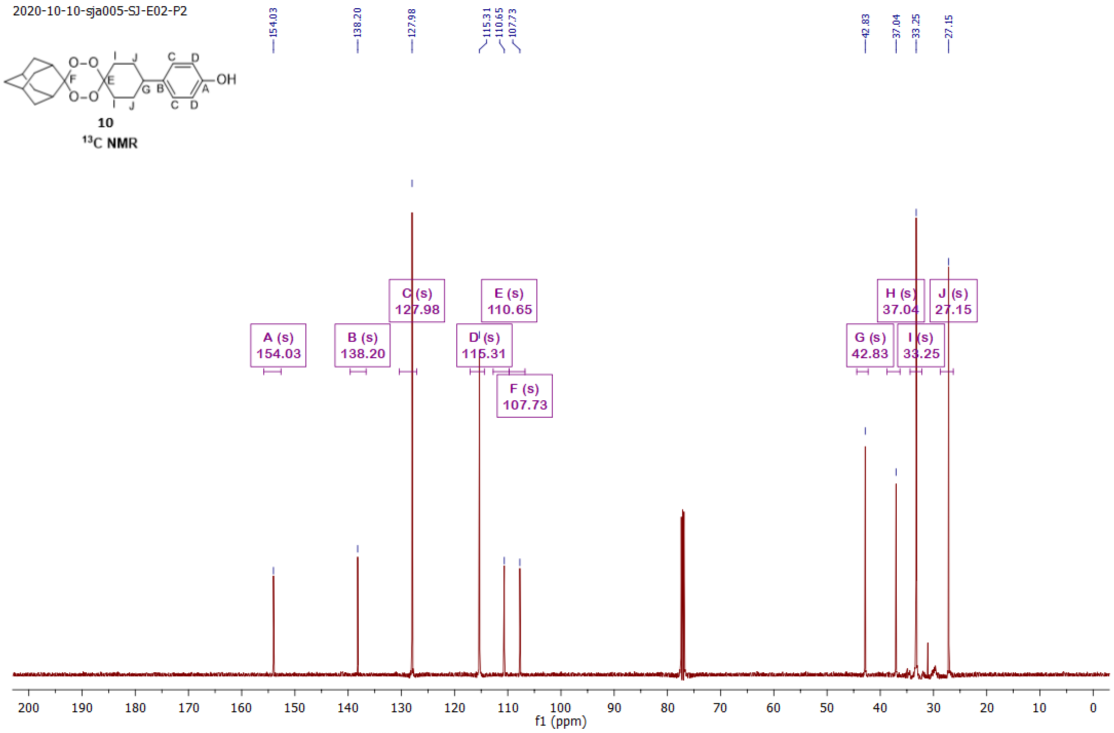

(1*r*,3*r*,5*r*,7*r*)-4''-(4-(4-bromobutoxy)phenyl)dispiro[adamantane-2,3'-[1,2,4,5]tetraoxane-6',1''-cyclohexane] (11)

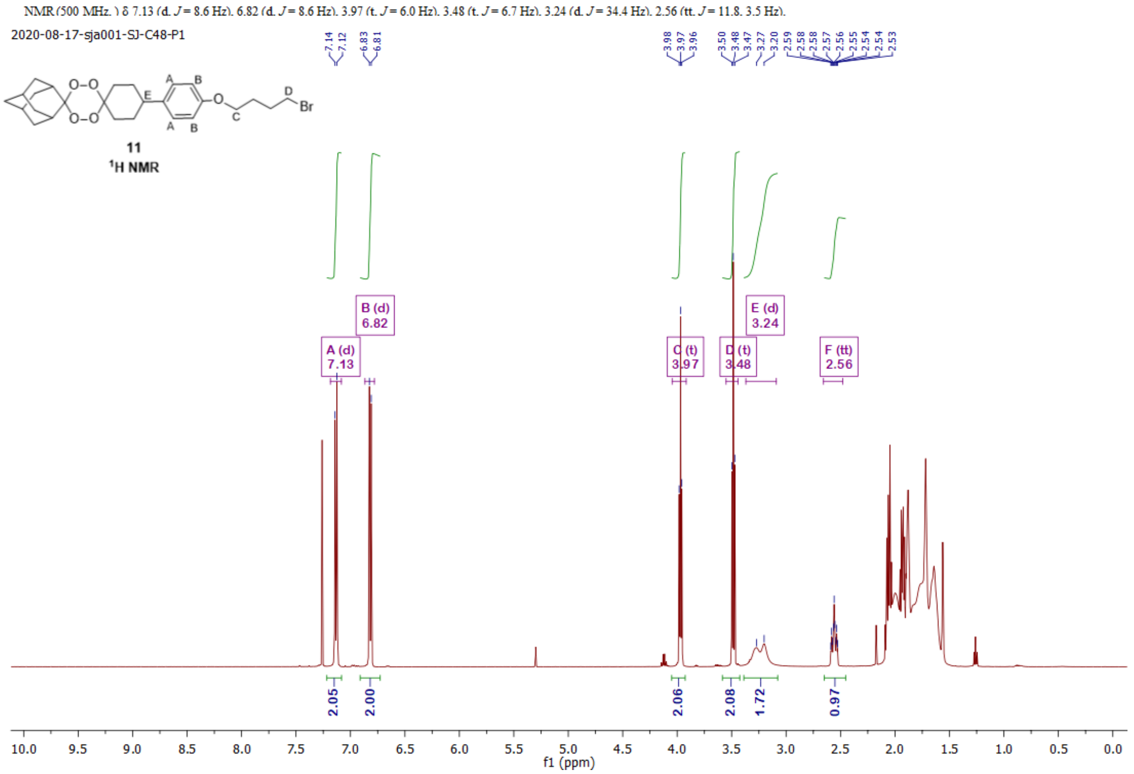

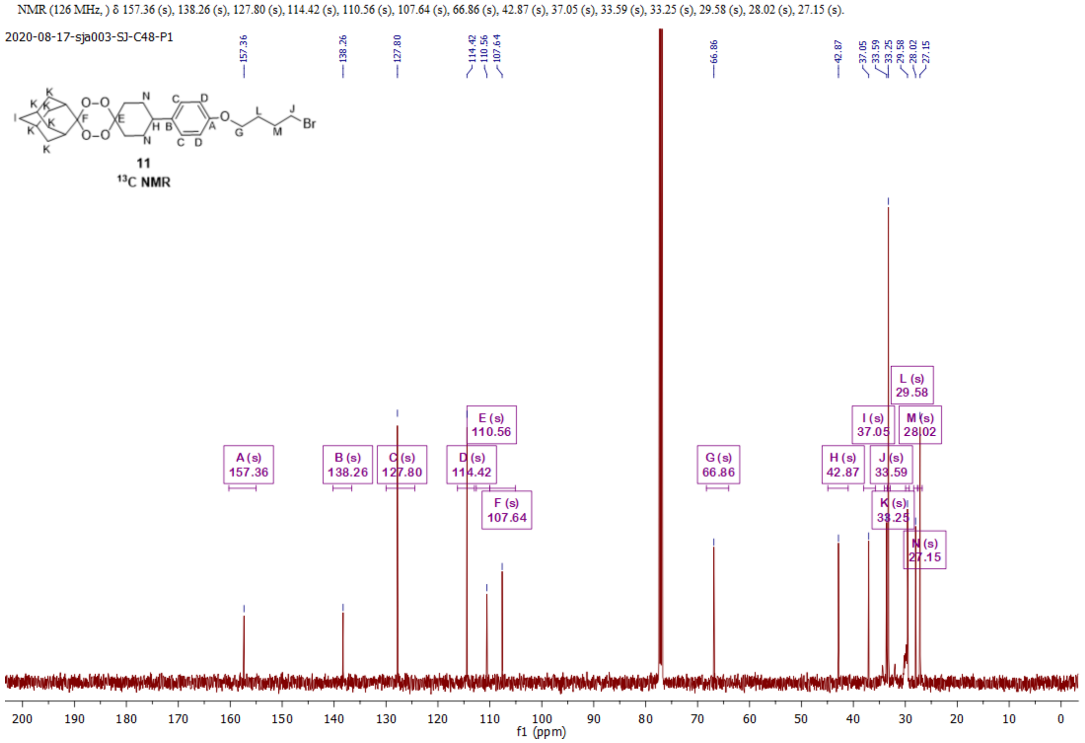

N^1-(4-(4-((1*r*,3*r*,5*r*,7*r*)-dispiro[adamantane-2,3'-[1,2,4,5]tetraoxane-6',1''-cyclohexan]-4''-yl) phenoxy)butyl)-N^4-(6-methoxy-5-phenoxyquinolin-8-yl)pentane-1,4-diamine (12)

NMR (500 MHz,) δ 8.52 (ddd, *J* = 4.1, 1.6, 0.5 Hz), 8.04 (ddd), 7.24 – 7.19 (m), 7.06 (d, *J* = 8.7 Hz), 6.95 (td, *J* = 7.3, 0.5 Hz), 6.85 (d, *J* = 7.8 Hz), 6.72 (d, *J* = 8.4 Hz), 6.54 (s), 3.90 (s), 3.86 – 3.77 (m), 3.72 (dd, *J* = 12.5, 6.3 Hz), 3.22 (d, *J* = 38.2 Hz), 2.98 – 2.81 (m), 2.51 (tt, *J* = 11.6, 3.5 Hz), 2.08 – 1.59 (m), 1.30 (d, *J* = 6.3 Hz).

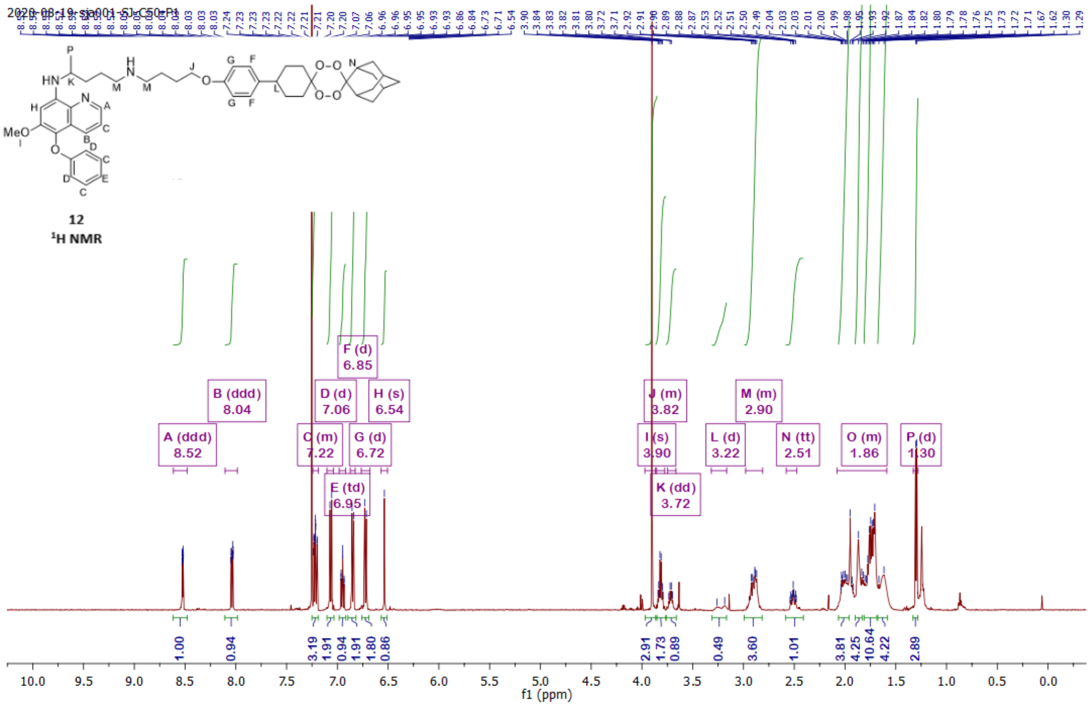

NMR (126 MHz,) δ 159.41 (s), 157.11 (s), 150.53 (s), 145.05 (s), 142.70 (s), 138.38 (s), 133.96 (s), 129.96 (s), 129.56 (s), 127.79 (s), 125.06 (s), 124.69 (s), 122.14 (s), 121.59 (s), 114.93 (s), 114.43 (s), 110.55 (s), 107.62 (s), 94.80 (s), 67.19 (s), 57.22 (s), 48.50 (s), 48.26 (s), 47.88 (s), 42.83 (s), 37.04 (s), 34.41 (s), 33.24 (s), 29.78 (s), 27.15 (s), 26.70 (s), 24.10 (s), 20.72 (s).

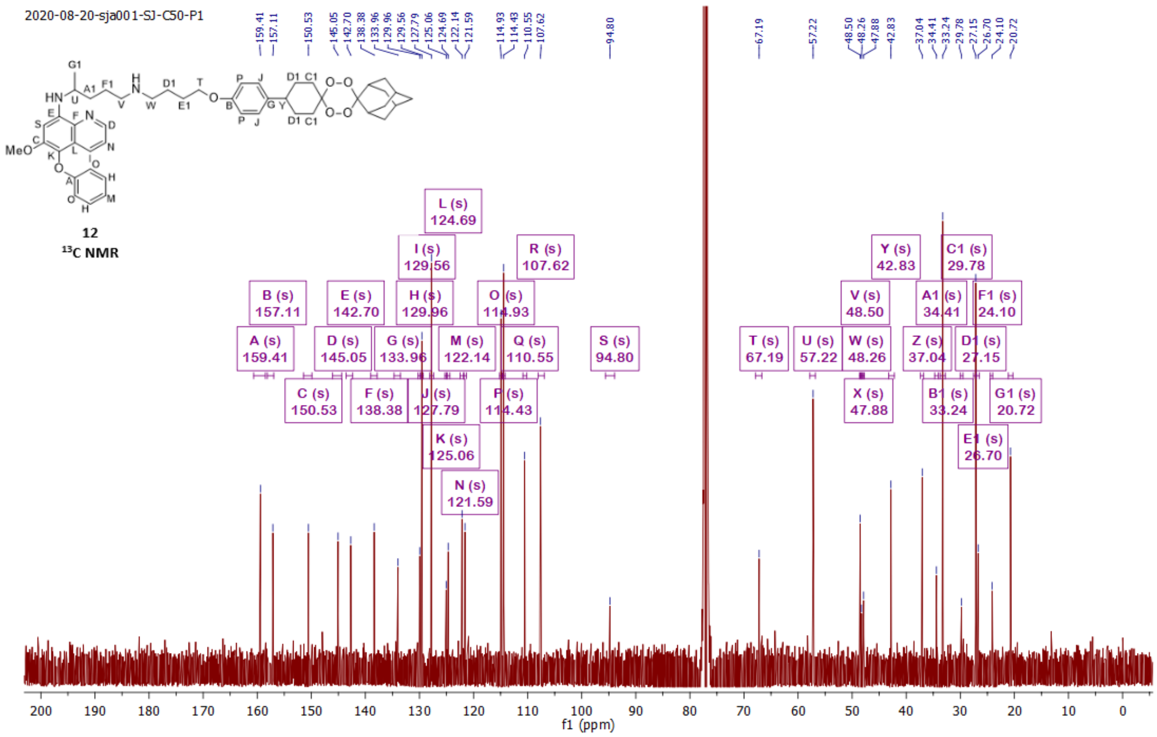

High resolution report

Analysis Name	D:\Data\customer\SJ C50 P1.d	Acquisition Date	9/9/2020 3:01:38 PM
Method	NaFormate_pos.m	Operator	Sutichai Ext: 3560
Sample Name	SJ C50 P1	Instrument	micrOTOF Bruker
		Calibrate by	Sodium Formate

Acquisition Parameter

Source Type	ESI	Ion Polarity	Positive	Set Nebulizer	0.3 Bar
Focus	Not active			Set Dry Heater	180 ?C
Scan Begin	100 m/z	Set Capillary	4500 V	Set Dry Gas	4.0 l/min
Scan End	2000 m/z	Set End Plate Offset	-500 V	Set Divert Valve	Source

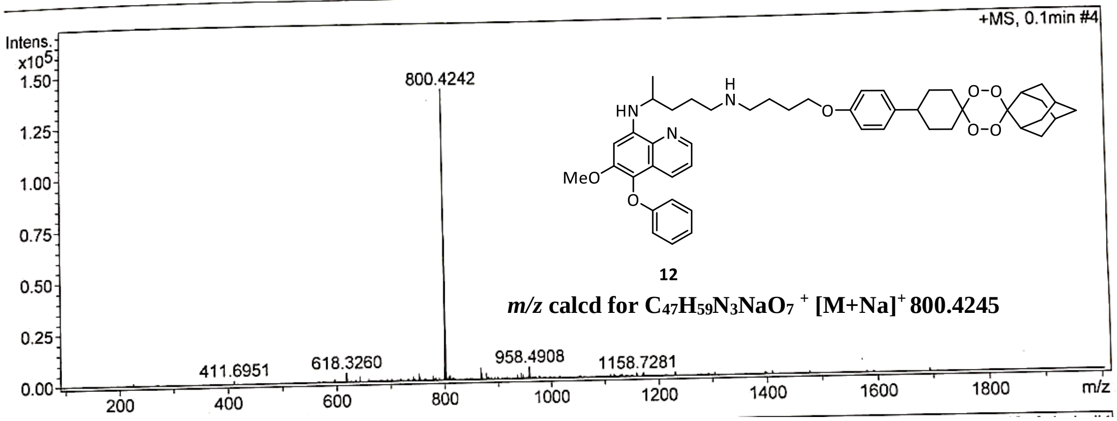

12

m/z **calcd for $C_{47}H_{59}N_3NaO_7{}^+$ $[M+Na]^+$ 800.4245**

Primary data for heme polymerization inhibition assay

Control	T	T-
1	0.5446	1.3240
2	0.5361	1.3336
3	0.5828	1.3263
Average	0.5545	1.3280

PQ	conc	1	2	3	HCF1	HCF2	HCF3	%Inh1	%Inh2	%Inh3		
	400	1.1201	1.1807	1.1501	0.269	0.190	0.230	73.12	80.96	77.01		
	200	0.9117	0.8333	0.8610	0.538	0.640	0.604	46.18	36.04	39.63		
	100	0.7047	0.7321	0.7060	0.806	0.770	0.804	19.42	22.96	19.59		
	50	0.6525	0.5004	0.5950	0.873	1.070	0.948	12.67	-7.00	5.24		
	10	0.4823	0.6216	0.5831	1.093	0.913	0.963	-9.33	8.67	3.70		
	2	0.5384	0.6796	0.5908	1.021	0.838	0.953	-2.08	16.17	4.69		
							IC50	312.8	309.9	336.8	319.8	Average
											12.0	SD

7a	conc	1	2	3	HCF1	HCF2	HCF3	%Inh1	%Inh2	%Inh3		
R = H	400	1.3420	1.3328	1.2949	-0.018	-0.006	0.043	101.81	100.63	95.73		
	200	1.2442	1.2371	1.2369	0.108	0.118	0.118	89.17	88.25	88.23		
	100	0.9465	1.0180	0.9378	0.493	0.401	0.505	50.68	59.92	49.55		
	50	0.7077	0.7989	0.7428	0.802	0.684	0.757	19.81	31.60	24.35		
	10	0.5876	0.6523	0.6299	0.957	0.874	0.903	4.28	12.65	9.75		
	2	0.5604	0.6189	0.5991	0.992	0.917	0.942	0.76	8.32	5.77		
							IC50	104.9	96.0	103.9	101.6	Average
											4.1	SD

7b	conc	1	2	3	HCF1	HCF2	HCF3	%Inh1	%Inh2	%Inh3		
R = OMe	400	1.2860	1.2564	1.3151	0.054	0.093	0.017	94.57	90.75	98.34		
	200	1.0747	1.0745	1.1075	0.328	0.328	0.285	67.25	67.23	71.49		
	100	0.8648	0.8585	0.8842	0.599	0.607	0.574	40.12	39.30	42.63		
	50	0.6724	0.7042	0.6810	0.848	0.806	0.837	15.24	19.36	16.35		
	10	0.5933	0.6127	0.6461	0.950	0.925	0.882	5.02	7.53	11.84		
	2	0.5750	0.5950	0.5688	0.974	0.948	0.982	2.65	5.23	1.85		
							IC50	162.1	159.6	156.7	159.5	Average
											2.2	SD

7c	conc	1	2	3	HCF1	HCF2	HCF3	%Inh1	%Inh2	%Inh3		
R = Br	400	1.2540	1.2423	1.2580	0.096	0.111	0.091	90.44	88.93	90.95		
	200	1.0445	1.0467	1.0307	0.367	0.364	0.384	63.35	63.64	61.57		
	100	0.7978	0.8029	0.8502	0.685	0.679	0.618	31.46	32.12	38.23		
	50	0.6525	0.6955	0.6376	0.873	0.818	0.893	12.67	18.23	10.74		
	10	0.6204	0.5995	0.6111	0.915	0.942	0.927	8.52	5.82	7.32		
	2	0.6135	0.6058	0.5650	0.924	0.934	0.986	7.63	6.63	1.36		
							IC50	170.0	180.6	174.8	175.2	Average
											4.3	SD

7d	conc	1	2	3	HCF1	HCF2	HCF3	%Inh1	%Inh2	%Inh3		
R = Cl	400	1.3014	1.3228	1.2608	0.034	0.007	0.087	96.57	99.33	91.32		
	200	1.1881	1.1676	1.1406	0.181	0.207	0.242	81.92	79.27	75.78		
	100	0.8563	0.9389	0.8664	0.610	0.503	0.597	39.02	49.70	40.32		
	50	0.7722	0.7133	0.7552	0.719	0.795	0.741	28.15	20.53	25.95		
	10	0.6479	0.6889	0.6284	0.879	0.826	0.905	12.08	17.37	9.55		
	2	0.6147	0.6029	0.5760	0.922	0.937	0.972	7.78	6.26	2.78		
							IC50	135.4	124.7	136.6	132.2	Average
											5.3	SD

7e	conc	1	2	3	HCF1	HCF2	HCF3	%Inh1	%Inh2	%Inh3		
R = CN	400	1.1611	1.2140	1.0931	0.216	0.147	0.304	78.42	85.27	69.64		
	200	0.9587	0.9868	0.9039	0.477	0.441	0.548	52.26	55.89	45.17		
	100	0.7875	0.7915	0.6884	0.699	0.694	0.827	30.12	30.64	17.31		
	50	0.7039	0.6810	0.7223	0.807	0.837	0.783	19.32	16.35	21.70		
	10	0.5837	0.6538	0.6274	0.962	0.872	0.906	3.78	12.84	9.43		
	2	0.6189	0.5844	0.6159	0.917	0.961	0.921	8.32	3.87	7.94		
							IC50	291.1	269.3	297.1	285.8	Average
											11.9	SD

7f	conc	1	2	3	HCF1	HCF2	HCF3	%Inh1	%Inh2	%Inh3		
R = F	400	1.2465	1.2646	1.3609	0.105	0.082	-0.043	89.47	91.81	104.26		
	200	1.0774	1.1136	1.1214	0.324	0.277	0.267	67.61	72.28	73.29		
	100	0.8393	0.8052	0.8321	0.632	0.676	0.641	36.82	32.41	35.89		
	50	0.7010	0.7465	0.6951	0.811	0.752	0.818	18.94	24.82	18.18		
	10	0.6656	0.6128	0.6293	0.856	0.925	0.903	14.36	7.54	9.67		
	2	0.6011	0.5685	0.6436	0.940	0.982	0.885	6.02	1.81	11.52		
							IC50	155.3	170.6	175.4	167.1	Average
											8.6	SD

7g	conc	1	2	3	HCF1	HCF2	HCF3	%Inh1	%Inh2	%Inh3		
R = CF3	400	1.2701	1.3094	1.3367	0.075	0.024	-0.011	92.52	97.60	101.13		
	200	1.2291	1.2514	1.2888	0.128	0.099	0.051	87.22	90.10	94.94		
	100	0.9072	0.9439	0.9565	0.544	0.497	0.480	45.60	50.34	51.98		
	50	0.7905	0.8434	0.7615	0.695	0.627	0.732	30.51	37.35	26.76		
	10	0.6662	0.7085	0.6369	0.856	0.801	0.894	14.44	19.91	10.65		
	2	0.5617	0.6305	0.6619	0.991	0.902	0.861	0.93	9.83	13.89		
							IC50	107.6	114.2	107.2	109.7	Average
											3.2	SD

7h	conc	1	2	3	HCF1	HCF2	HCF3	%Inh1	%Inh2	%Inh3		
R = CONH2	400	1.2292	1.2707	1.3532	0.128	0.074	-0.033	87.23	92.60	103.26		
	200	1.0705	1.1566	1.2061	0.333	0.222	0.158	66.71	77.84	84.25		
	100	0.8632	0.9015	0.8701	0.601	0.551	0.592	39.91	44.86	40.80		
	50	0.7271	0.7882	0.7887	0.777	0.698	0.697	22.32	30.21	30.28		
	10	0.6189	0.6897	0.6984	0.917	0.825	0.814	8.33	17.48	18.60		
	2	0.6080	0.5767	0.6640	0.931	0.971	0.858	6.92	2.87	14.16		
							IC50	153.6	161.7	146.0	153.8	Average
											6.4	SD

12	conc	1	2	3	HCF1	HCF2	HCF3	%Inh1	%Inh2	%Inh3		
conjugate	400	1.3771	1.2946	1.3063	-0.063	0.043	0.028	106.35	95.69	97.20		
	200	1.2526	1.2954	1.2334	0.098	0.042	0.122	90.25	95.79	87.78		
	100	1.1903	1.1218	1.0502	0.178	0.267	0.359	82.20	73.34	64.09		
	50	0.9274	0.9397	0.9501	0.518	0.502	0.489	48.21	49.80	51.14		
	10	0.7022	0.7176	0.6639	0.809	0.789	0.859	19.10	21.09	14.14		
	2	0.6519	0.6822	0.6232	0.874	0.835	0.911	12.59	16.51	8.88		
							IC50	64.5	64.3	71.8	66.9	Average
											3.5	SD

CQ	conc	1	2	3	HCF1	HCF2	HCF3	%Inh1	%Inh2	%Inh3		
	400	1.3721	1.2840	1.3079	-0.057	0.057	0.026	105.70	94.31	97.41		
	200	1.2998	1.1170	1.2177	0.036	0.273	0.143	96.36	72.73	85.74		
	100	1.2104	1.1826	1.0987	0.152	0.188	0.296	84.80	81.20	70.36		
	50	0.9397	0.8390	1.0169	0.502	0.632	0.402	49.80	36.78	59.78		
	10	0.7031	0.7685	0.7640	0.808	0.723	0.729	19.21	27.67	27.09		
	2	0.6704	0.6490	0.6412	0.850	0.878	0.888	14.98	12.22	11.21		
							IC50	61.3	59.6	62.8	61.2	Average
											1.3	SD
